# The protection of mesenchymal stem cells in metabolic reprogramming and endothelial-mesenchymal transition in diabetic aortas

**DOI:** 10.1093/stcltm/szaf077

**Published:** 2026-01-13

**Authors:** Mingying Ling, Jingxian He, Xu Jia, Na Yu, Yiping Song, Xuehui Li, Congmin Tang, Wenzhuo Yu, Han Qiao, Chenglong Zhang, Zhen Zhang, Tianmin Ma, Chuanli Zhao, Yanqiu Xing

**Affiliations:** Department of Geriatric Medicine, Laboratory of Gerontology and Anti-aging Research, Jinan Clinical Research Center for Geriatric Medicine, Qilu Hospital of Shandong University, Jinan, Shandong 250012, China; Department of Geriatric Medicine, Laboratory of Gerontology and Anti-aging Research, Jinan Clinical Research Center for Geriatric Medicine, Qilu Hospital of Shandong University, Jinan, Shandong 250012, China; Institute of Basic Medical Sciences, Qilu Hospital of Shandong University, Jinan, Shandong 250012, China; Department of Geriatric Medicine, Laboratory of Gerontology and Anti-aging Research, Jinan Clinical Research Center for Geriatric Medicine, Qilu Hospital of Shandong University, Jinan, Shandong 250012, China; Shandong Precision Medicine Engineering Laboratory of Bacterial Anti-tumor Drugs, Jinan, Shandong 250101, China; College of Clinical Medicine, Shandong University, 250012 Jinan, Shandong, China; Department of Geriatric Medicine, Laboratory of Gerontology and Anti-aging Research, Jinan Clinical Research Center for Geriatric Medicine, Qilu Hospital of Shandong University, Jinan, Shandong 250012, China; Department of Geriatric Medicine, Laboratory of Gerontology and Anti-aging Research, Jinan Clinical Research Center for Geriatric Medicine, Qilu Hospital of Shandong University, Jinan, Shandong 250012, China; Department of Geriatric Medicine, Laboratory of Gerontology and Anti-aging Research, Jinan Clinical Research Center for Geriatric Medicine, Qilu Hospital of Shandong University, Jinan, Shandong 250012, China; Institute of Basic Medical Sciences, Qilu Hospital of Shandong University, Jinan, Shandong 250012, China; Department of Geriatric Medicine, Laboratory of Gerontology and Anti-aging Research, Jinan Clinical Research Center for Geriatric Medicine, Qilu Hospital of Shandong University, Jinan, Shandong 250012, China; Institute of Basic Medical Sciences, Qilu Hospital of Shandong University, Jinan, Shandong 250012, China; Department of Geriatric Medicine, Laboratory of Gerontology and Anti-aging Research, Jinan Clinical Research Center for Geriatric Medicine, Qilu Hospital of Shandong University, Jinan, Shandong 250012, China; Institute of Basic Medical Sciences, Qilu Hospital of Shandong University, Jinan, Shandong 250012, China; Department of Geriatric Medicine, Laboratory of Gerontology and Anti-aging Research, Jinan Clinical Research Center for Geriatric Medicine, Qilu Hospital of Shandong University, Jinan, Shandong 250012, China; Institute of Basic Medical Sciences, Qilu Hospital of Shandong University, Jinan, Shandong 250012, China; Department of Geriatric Medicine, Laboratory of Gerontology and Anti-aging Research, Jinan Clinical Research Center for Geriatric Medicine, Qilu Hospital of Shandong University, Jinan, Shandong 250012, China; Service Improvement and Innovation, Te Whatu Ora Health New Zealand, Auckland, 2014, New Zealand; Department of Geriatric Medicine, Laboratory of Gerontology and Anti-aging Research, Jinan Clinical Research Center for Geriatric Medicine, Qilu Hospital of Shandong University, Jinan, Shandong 250012, China; Department of Geriatric Medicine, Laboratory of Gerontology and Anti-aging Research, Jinan Clinical Research Center for Geriatric Medicine, Qilu Hospital of Shandong University, Jinan, Shandong 250012, China

**Keywords:** diabetes, mesenchymal stem cells, vascular remodeling, cell type proteomics, metabolomics, metabolic reprogramming

## Abstract

Vascular remodeling, a precursor to atherosclerosis and coronary heart disease, is associated with high morbidity and mortality in individuals with diabetes. The roles of endothelial-mesenchymal transition (EndMT) and human umbilical cord mesenchymal stem cells (hUCMSCs) in this process remain unclear. In this study, we used db/db mice as a diabetic model to investigate the effect of hUCMSCs on metabolic reprogramming and vascular remodeling. We analyzed serum markers, tissue morphology, metabolomics, and endothelial cell-specific proteomics. The results demonstrated that vascular remodeling and EndMT were exacerbated in diabetes and alleviated by hUCMSCs. Metabolomic analysis identified 209 altered metabolites. Most metabolic intermediates were increased, while anti-inflammatory metabolites such as arachidonoyl ethanolamide and sphingosine were decreased in the diabetic state. Treatment with hUCMSCs restored these metabolites to near-normal levels, thereby improving metabolic reprogramming and the vascular microenvironment. Correspondingly, endothelial cell proteomics revealed increased levels of glycolytic enzymes, inflammatory factors, and EndMT markers, including mitogen-activated protein kinase kinase kinase 20 (Map3k20), disintegrin and metalloproteinase domain-containing protein 10 (Adam10), and integrin alpha-8 (Itga8), in diabetes; hUCMSC treatment downregulated these factors. Notably, KEGG and protein–protein interaction analyses indicated that hUCMSCs inhibited the Tgfb1i1/Rock1 axis within the TGF-beta pathway, which drives EndMT. We further verified the expression of these proteins through endothelial immunofluorescent co-staining and confirmed the role of Rock1 in high glucose-induced EndMT *in vitro*. This study elucidates a potential molecular mechanism and a therapeutic strategy for early atherosclerosis in diabetes and provides a foundation for evaluating endothelial states *in vivo*.

## Graphical abstract

**Graphical Abstract:**
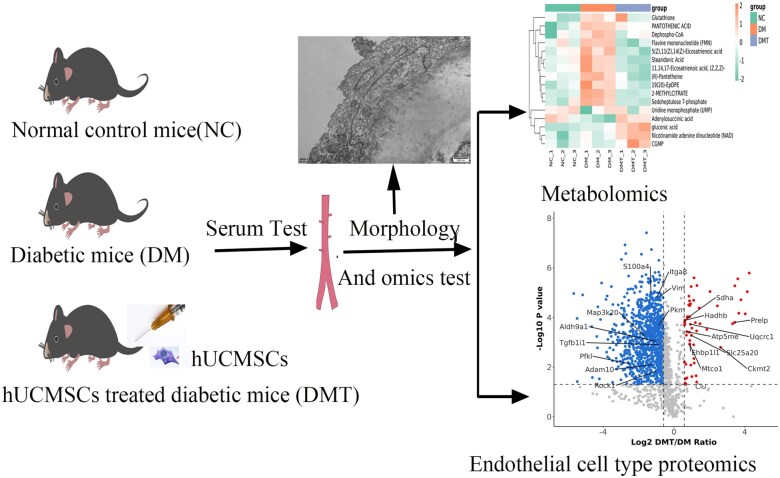

Significance statement Metabolic disorders and alterations in cell phenotype are increasingly recognized as factors in diabetic atherosclerosis. This study elucidates the protective role of human umbilical cord mesenchymal stem cells (hUCMSCs) in the metabolic reprogramming, inflammatory microenvironment, and endothelial-mesenchymal transition within diabetic aortas. By integrating comprehensive metabolomic and endothelial cell-specific proteomic analyses, this work establishes a connection between hUCMSC treatment and the remodeling of the vascular microenvironment in diabetes.

## Introduction

Diabetic vascular remodeling is a primary cause of atherosclerosis and coronary heart disease, and represents one of the most severe complications of diabetes.^1^ Increasing attention has focused on the role of cell phenotype transformations in atherosclerosis.^2^ However, understanding of the specific stimuli that drive these cellular changes in diabetes and their significance for vascular remodeling remains limited. Furthermore, while current therapies can delay the progression of diabetic vascular remodeling, effective interventional strategies are still lacking. Therefore, studies to identify underlying mechanisms and potential treatments are urgently needed.

Endothelial-mesenchymal transition (EndMT) is a pathological process in which endothelial cells transform into mesenchymal phenotypes in response to triggers such as hyperglycemia and inflammatory factors.^3^ Aortic remodeling, accompanied by accumulation of extracellular matrix components including collagens and smooth muscle actin (SMA), can promote atherosclerosis and may lead to lumen occlusion. During EndMT, the expression of specific endothelial markers like platelet endothelial cell adhesion molecule-1 (CD31) decreases, while mesenchymal markers such as transgelin (Tagln/SM22) and protein S100-A4 (S100a4) increase. Abnormal EndMT has been observed in cardiovascular diseases including atherosclerosis and cardiac fibrosis.^4^ Consequently, investigating the mechanisms and potential interventions of EndMT represents a promising research direction. However, progress in this area has been constrained by the lack of robust animal models for establishing causality, as well as insufficient understanding of the precise functional and molecular pathways of EndMT across different disease contexts.

Mesenchymal stem cells (MSCs) are characterized by their multipotent differentiation capability, self-renewal capacity, immunomodulatory properties, low immunogenicity, and roles in tissue regeneration and anti-inflammatory responses. MSCs from different sources exhibit distinct molecular phenotypes and may possess different functional features. Current MSC-based interventions primarily focus on cell transplantation. Human umbilical cord mesenchymal stem cells (hUCMSCs) have attracted increasing attention for clinical translation due to their ready availability, high proliferative potential, and low immunogenicity.^5^ Recent studies have demonstrated that hUCMSCs are a safe and effective therapy for lung damage and impaired lung function in patients with COVID-19, suggesting their potential as a promising approach for managing diabetic complications.^6^^,^^7^ However, the role and mechanism of hUCMSCs in diabetic vascular remodeling, particularly in endothelial-mesenchymal transition (EndMT), remain unclear.

Proteins act as key executors and regulators of cellular activities. In cell-type-specific proteomics, target cells are isolated from tissues using enzymatic digestion and sorted into individual wells. This approach allows for cell-type identification through downstream analysis while preserving the disease context present *in vivo*, thereby adding a critical dimension to proteomic studies.^8^ Meanwhile, metabolomics can profile the metabolic environment within the arteries of diabetic mice. The integrated analysis of metabolomic and single-cell proteomic data is transforming our understanding of cell biology in both health and disease.^9^

Therefore, we hypothesized that hUCMSCs ameliorate aortic remodeling in diabetes primarily through modulating EndMT, as revealed by endothelial cell-specific proteomics and metabolomics. In this study, db/db mice were used as a model of type 2 diabetes mellitus (T2DM) to investigate its complications. A comprehensive analysis was conducted to clarify the potential therapeutic effects and underlying mechanisms of hUCMSCs in diabetic aortic remodeling. Transmission electron microscopy (TEM) and immunohistochemistry (IHC) were employed for pathological examination to assess disease progression and identify potential biomarkers.

## Methods

### Preparation of hUCMSCs

HUCMSCs were isolated and characterized as previously described in our study.^10^ Briefly, fresh umbilical cords were obtained from consenting mothers at Qilu Hospital of Shandong University, China, and processed immediately. Cells from passages 3 to 7 (F3-F7) were used for all experiments. HUCMSCs were identified according to the criteria established by the International Society for Cellular Therapy, which included: (1) multilineage differentiation potential assessed using Alizarin Red S (osteogenesis), Oil Red O (adipogenesis), and Alcian Blue (chondrogenesis) staining, and (2) surface marker profiling by flow cytometry.^11^ The experimental protocol was approved by the Ethics Committee of Shandong University. A detailed description of the isolation and culture process is provided in the Supplementary Materials.

### Animals

Male C57BLKS/J db/db and db/m mice (*n* = 18, 8-week old) were supplied by Beijing Vital River Laboratory Animal Technology Co., Ltd. All procedures were conducted in compliance with the Animal Management Rules of the Chinese Ministry of Health (Document No. 55, 2001) and were approved by the Animal Care Committee of Shandong University. After a 6-week acclimatization period, db/m mice were assigned to a normal control group (NC, *n* = 6). The db/db mice were randomly allocated to either a saline-treated diabetic group (DM, *n* = 6) or an hUCMSCs-treated group (DMT, *n* = 6). Mice in the DMT group received 1 × 10^5^ hUCMSCs diluted in normal saline via tail vein injection once per week for 6 weeks. All mice were monitored from 8 to 22 weeks of age without administration of any hypoglycemic agents. Body weight (BW) and fasting blood glucose (FBG) levels were measured regularly. After an overnight fast, all mice were euthanized under sodium pentobarbital anesthesia. Aortas were dissected, stored at −80 °C, and subsequently used for analysis.

### Examination of serum samples

Serum samples were obtained from orbital venous blood following centrifugation at 3000 rpm for 30 min. The serum concentration of glycosylated serum protein (GSP) was measured by chemiluminescence assay using an automatic biochemical analyzer (Hitachi, Tokyo, Japan) in accordance with commercial test kit instructions. Serum levels of interleukin-6 (IL-6) and interleukin-10 (IL-10) were quantified using commercially available enzyme-linked immunosorbent assay (ELISA) kits from Wuhan Gene Beauty Biotechnology.

### Morphological and immunohistochemical staining

Thoracic aortas were fixed in 4% paraformaldehyde, embedded in paraffin, and sectioned at 5 μm thickness. Sections were stained with hematoxylin and eosin (H&E) and Sirius red. For immunohistochemistry, sections were treated with 0.3% Triton X-100 in 0.1 M PBS, incubated with primary antibodies overnight at 4 °C, followed by incubation with secondary antibodies for 2 h at room temperature. Morphometric analysis was performed using AxioVision Rel 4.8.2 software (Carl Zeiss, Germany), and image analysis was conducted with Image-Pro Plus 6.0 (Media Cybernetics).

For transmission electron microscopy, aortic segments were fixed in 3% glutaraldehyde in cacodylate buffer for 2 h at 4 °C, then post-fixed in 1% osmium tetroxide in phosphate buffer for 1 h. Ultrathin sections were stained with uranyl acetate and lead citrate and examined using a JEOL 1200 EX transmission electron microscope (JEOL Ltd, Japan).

### Flow cytometry and endothelial cell sorting

Aortic tissues were minced into 0.5 mm^2^ pieces and digested with an enzyme solution. The resulting cell pellet was resuspended in 100 μL of 1× PBS containing 0.04% BSA. Cell viability, assessed by trypan blue exclusion, exceeded 90%. The single-cell suspension was stained with PE-conjugated CD31 antibody on ice for 20 min and sorted using a FACSAria™ III Cell Sorter (BD Biosciences). FACS events were selected through sequential gating. A detailed description of the procedure is provided in the Supplementary Materials.

### Untargeted metabolomics

Aortic samples (5 mg) were homogenized in 50 μL of H_2_O. Metabolite extraction was performed by adding 200 μL of methanol/acetonitrile (MeOH/ACN, 1:1, v/v), followed by vortexing and sonication. The supernatant was collected for LC/MS analysis and stored at −80 °C. Mass spectrometry was conducted using a Triple TOF 6600 system (AB/SCIEX, Framingham, MA, USA) coupled to a 1290 Infinity Ultra-High Performance Liquid Chromatography system (Agilent Technologies, Palo Alto, CA, USA). The detailed procedure is available in the Supplementary materials.

### Proteomic measurements

Label-free quantitative proteomics of sorted endothelial cells was performed using timsTOF Pro mass spectrometry (Bruker Daltonics, Bremen, Germany) with trapped ion mobility separation (TIMS) and parallel accumulation-serial fragmentation (PASEF). This 4D-proteomics approach incorporated ion mobility, *m*/*z*, retention time, and intensity dimensions for protein identification and quantification. The workflow included sample preparation, mass spectrometric analysis, and data processing. A detailed description of the procedure is provided in the Supplementary materials.

### Integrated metabolites and proteins analysis

Integrated metabolite-protein networks were constructed and annotated for enriched cellular and molecular functions using Cytoscape software version 3.4.0 (http://www.cytoscape.org/). Differentially abundant proteins and metabolites were consolidated into a single matrix using *Z*-score normalization.

### Endothelial cell culture and treatments

The human endothelial cell line EA.hy926 (ATCC) was cultured in DMEM supplemented with 10% (v/v) fetal bovine serum, 100 U/mL penicillin, and 100 μg/mL streptomycin at 37 °C in a humidified 5% CO_2_ incubator. Culture medium was replaced every 48 h, and cells were subcultured at 80% confluence. After seeding in 6-well plates (1 × 10^6^ cells/well) for 24 h, the medium was replaced, and cells were divided into four experimental groups: normal control (NC, 5.6 mM glucose), osmotic control (OC, 5.6 mM glucose + 24.5 mM mannitol), high glucose (HG, 30 mM glucose), and high glucose treatment (HGT, 30 mM glucose + 10 μm fasudil). All groups were incubated for 24 h under their respective conditions.

### RNA extraction and real-time PCR

Total cellular RNA was extracted using TRIzol^®^ reagent (Life Technologies, Carlsbad, CA, USA) according to the manufacturer’s protocol, followed by reverse transcription. Quantitative real-time PCR was performed using the Bio-Rad iQ5 Gradient Real-Time PCR system (BioRad) with SYBR^®^ Green Master mix (Premix Ex Taq™ kit; TaKaRa Biotechnology). Relative quantification of target genes was calculated using the 2^-ΔΔCt^ method, with normalization to GAPDH expression and calibration to the normal control (NC) group. All experiments included 3-4 biological replicates and were independently repeated three times. Primer sequences are listed in Table S1.

### Statistical analysis

Statistical analyses were performed using SPSS version 22.0 (IBM, New York, USA). Quantitative data are expressed as mean ± standard deviation (SD). Multiple group comparisons were conducted using one-way analysis of variance (ANOVA) with Bonferroni’s *post hoc* test. Comparisons between two groups were performed using Student’s *t*-test. For multiple *t*-tests, *P*-values were adjusted using the Ryan–Holm step-down Bonferroni procedure. Pearson correlation analysis between proteins and metabolites was performed and visualized using the Pearson algorithm in R version 3.5.1 (The R Foundation for Statistical Computing, Vienna, Austria). A *P*-value of less than .05 was considered statistically significant.

## Results

### HUCMSCs regulated blood glucose and inflammation of db/db mice

The experimental design, including combined analytical approaches and animal treatments, is summarized in the graphical abstract. The hUCMSCs were acquired and characterized according to the International Society for Cellular Therapy criteria (Figure S1).^11^

**Figure 1.**
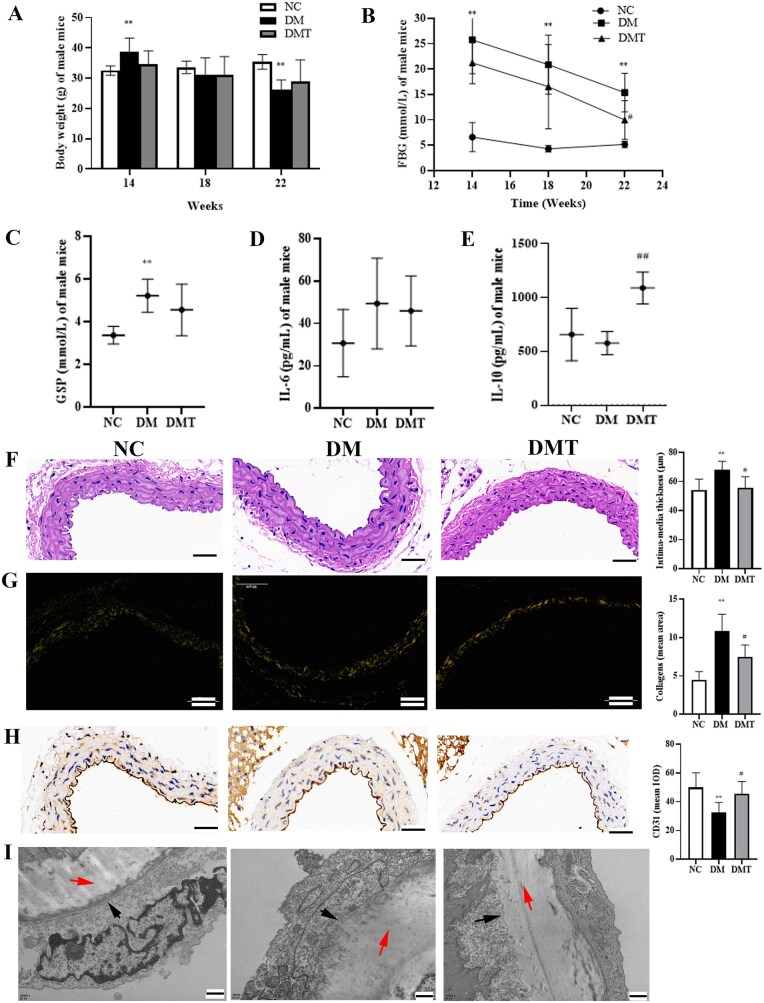
Effects of hUCMSCs on general variables and inhibition of aortic remodeling. (A) Body weight (BW), (B) fasting blood glucose (FBG), and (C) glycosylated serum protein (GSP) levels in mice. (D) Interleukin-6 (IL-6) and (E) interleukin-10 (IL-10) levels in mice. (F) Hematoxylin and eosin (H&E) staining, (G) Sirius red staining of collagen, and (H) immunohistochemical staining of CD31 in mouse aortas (×400; scale bars = 50 μm). (I) Representative transmission electron micrographs of the intima–media layer; black arrows indicate the basal membrane, red arrows indicate the internal elastic lamina (×30 000; scale bars = 500 nm). Data were analyzed by one-way ANOVA with Bonferroni’s *post hoc* test. DM, untreated db/db group; DMT, hUCMSC-treated db/db group; IOD, integrated optical density; NC, db/m control group. **P < .01 vs NC group; #*P *< .05, ##*P *< .01 vs DM group (Bonferroni-adjusted).

Throughout the study, mice were maintained without hypoglycemic medication. At 14 weeks, BW of db/db mice was significantly higher than that of db/m mice. By 18 and 22 weeks, BW in diabetic mice gradually decreased, while hUCMSCs treatment showed no significant effect on this trend (Figure 1A). Fasting blood glucose (FBG) levels in db/db mice were significantly elevated compared to db/m mice at 14, 18, and 22 weeks (*P* < .05). hUCMSCs administration significantly reduced FBG in diabetic mice at 22 weeks (*P* < .05; Figure 1B). Furthermore, glycosylated serum protein (GSP) levels were significantly higher in the db/db group compared to db/m mice (*P* < .01), but no significant difference was observed between the DMT and DM groups (*P* > .05; Figure 1C). Interleukin-6 (IL-6) levels showed no significant differences among the three groups (*P* > .05; Figure 1D). In contrast, interleukin-10 (IL-10) levels decreased in the DM group compared to the NC group, and hUCMSCs treatment restored IL-10 levels in the DMT group (Figure 1E and Table S2).

### HUCMSCs ameliorated aortic remodeling in db/db mice

H&E staining revealed aortic remodeling in the DM group, characterized by increased endothelial impairment and vascular smooth muscle cell (VSMC) proliferation compared to the NC group. The aortic walls were thicker and exhibited a disorganized, loose structure (Figure 1F). Collagen deposition was increased (Figure 1G), while expression of the endothelial marker CD31 was reduced (Figure 1H). Co-staining analysis demonstrated the presence of mesenchymal markers S100A4 and Transgelin in aortic endothelial cells (ECs) of the DM group (Figure S2), suggesting that endothelial-mesenchymal transition (EndMT) contributes to diabetic aortic remodeling. Notably, hUCMSCs treatment in the DMT group ameliorated endothelial damage, suppressed EndMT, and improved aortic remodeling, with results approaching those observed in the NC group (Figure 1F-H and Table S3).

According to the results of transmission electron microscopy of the aortas, which was used to more directly present the endothelial structure, aortic ECs in the NC group were flattened with little interstitial matrix, the basal membrane was intact, and the internal elastic lamina appeared straight and of uniform thickness (Figure 1I). On the other hand, the aortic ECs of the DM group were enlarged, the internal elastic lamina widened and became unevenly thick and even ruptured. Furthermore, there was a clear proliferation of the matrix fibers. However, in the DMT group, the intervention of hUCMSCs reduced these ultrastructural abnormalities (Figure 1I).

### Metabolomic analysis

#### Metabolic signatures

**Figure 2.**
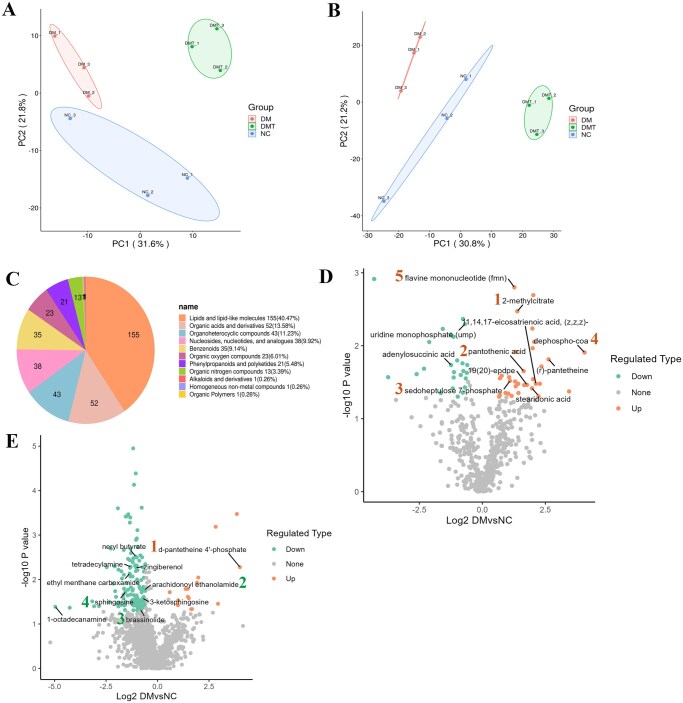
Multivariate statistical analysis of metabolomics and significant metabolite changes across groups. Principal component analysis (PCA) score plots from LC–MS-based metabolomic profiling of mouse aortas in (A) negative and (B) positive modes. (C) Classification of 383 identified metabolites. (D) Volcano plot of differentially abundant metabolites between the DM and NC groups in negative mode. Labeled metabolites: (1) 2-methylcitrate (FC = 2.62, *P *= .003), (2) pantothenic acid (FC = 3.11, *P *= .022), (3) sedoheptulose 7-phosphate (FC = 2.14, *P *= .030), (4) dephospho-CoA (FC = 16.63, *P *= .012), (5) flavin mononucleotide (FMN; FC = 2.41, *P *= .002); 11,14,17-eicosatrienoic acid (FC = 4.35, *P *= .033), stearidonic acid (FC = 3.92, *P *= .038), (R)-pantetheine (FC = 6.18, *P *= .015), uridine monophosphate (UMP; FC = 0.62, *P *= .005), adenylosuccinic acid (FC = 0.42, *P *= .018). (E) Volcano plot of differentially abundant metabolites between the DM and NC groups in positive mode. Labeled metabolites: (1) D-pantetheine 4'-phosphate (FC = 16.05, *P *= .005), (2) arachidonoyl ethanolamide (FC = 0.65, *P *= .017), (3) brassinolide (FC = 0.56, *P *= .048), (4) sphingosine (FC = 0.34, *P *= .019); ethyl menthane carboxamide (FC = 0.39, *P *= .007), neryl butyrate (FC = 0.48, *P *= .003), zingiberenol (FC = 0.49, *P *= .005), 1-octadecanamine (FC = 0.03, *P *= .041), tetradecylamine (FC = 0.41, *P *= .005), (±)-[R-(E)]-3-ketosphingosine (FC = 0.61, *P *= .027). All *P*-values are Bonferroni-adjusted. Twenty metabolites were significantly altered in the DM group compared to NC (10 in negative and 10 in positive mode). Numbers indicate key metabolites. DM, untreated db/db group; DMT, hUCMSC-treated db/db group; LC–MS, liquid chromatography–mass spectrometry; NC, db/m control group.

PCA score plots were shown in negative (Figure 2A) and positive (Figure 2B) modes for the normal control (NC, blue circles), diabetic (DM, red circles), and hUCMSCs-treated (DMT, green circles) groups. The close clustering of intra-group samples indicated stable, robust, and reliable data quality. Orthogonal partial least squares–discriminant analysis confirmed the model’s predictability, stability, and repeatability (Table S4). Figure S2 displayed representative LC–MS total ion chromatograms in positive and negative modes.

A total of 1787 metabolites were identified after data processing, of which 383 were further classified. Among the three groups, 504 and 1341 metabolites were detected in negative and positive modes, respectively. Lipids and lipid-like molecules represented the most abundant category, accounting for 40.47% of the classified metabolites, followed by organic acids and their derivatives at 13.58% (Figure 2C).

#### Differential metabolites analysis

A total of 209 metabolites (57 in negative and 152 in positive mode) were significantly altered in the DM group compared to the NC group, based on thresholds of *P *< .05, VIP ≥ 1.0, and FC ≥ 1.5. Of the 57 metabolites identified in negative mode, 26 were assigned to structural categories. Fifteen of these metabolites (26.32%) were classified as organic acids and derivatives, lipids and lipid-like molecules, or organic oxygen compounds. Among these 15 metabolites, the majority (10) were present at higher levels in the DM group than in the NC group. These included pantothenic acid, gluconic acid, and sedoheptulose 7-phosphate in organic oxygen compounds (3 of 5), (R)-pantetheine, glutathione, and 2-methylcitrate in organic acids and derivatives (3 of 5), 11,14,17-eicosatrienoic acid, stearidonic acid, 19(20)-EpDPE, and 5(Z),11(Z),14(Z)-eicosatrienoic acid in lipids and lipid-like molecules (4 of 5). Nucleosides, nucleotides, and analogues related to energy metabolism, such as dephospho-CoA, flavin mononucleotide (FMN), and nicotinamide adenine dinucleotide (NAD), were upregulated in the DM group compared to the NC group, whereas those related to nucleic acid metabolism decreased, such as uridine monophosphate (UMP), cGMP, and adenylosuccinic acid (Figure 2D and Table S5).

Thirty-four of the 152 metabolites that were altered in the DM group compared to the NC group could be further categorized in positive mode. Furthermore, 26 of the 152 metabolites (17.11%) were classified as organic nitrogen compounds (6 of 152), organic acids and derivatives (5 of 152), organic oxygen compounds (4 of 152), and lipids and lipid-like molecules (11 of 152). Additionally, the majority of them (22 out of 26) were lower in the DM group than in the NC group. Downregulated metabolites with various protective or anti-inflammatory effects, particularly on the vascular endothelium, included brassinolide, (E, E)-2,4-decadienoic isobutylamide, 1-palmitoyl-sn-glycero-3-phosphocholine, and 1-heptadecanoyl-sn-glycero-3-phosphocholine in lipids and lipid-like molecules (4 of 11), as well as arachidonoyl ethanolamide and sphingosine in organic nitrogen compounds (2 of 6). Moreover, a large number of other metabolites also declined in the DM group with similar roles, including ethyl menthane carboxamide, neryl butyrate, zingiberenol, 2-[octahydro-4,7-dimethyl-1-oxocyclopenta[c]pyran-3-yl]nepetalactam, and geranyl acetoacetate in lipids and lipid-like molecules (5 of 11); 1-octadecanamine, tetradecylamine, and xestoaminol C in organic nitrogen compounds (3 of 6); and (+/-)-[R-(E)]-3-ketosphingosine, (Z)-9-cycloheptadecen-1-one, and 5-isopropyl-8-methylnona-6,8-dien-2-one in organic oxygen compounds (3 of 4). On the other hand, the majority of metabolic intermediates associated with organic acids and their derivatives, such as glutathione, tyrosyl-aspartate, and D-pantetheine 4'-phosphate, increased (Figure 2E and Table S6).

Of the 209 metabolites altered in the DM group compared to the NC group, hUCMSC treatment in the DMT group significantly restored 127 metabolites to near-normal levels, including 29 in negative mode and 98 in positive mode. Specifically, hUCMSCs downregulated 27 metabolites that were upregulated in the DM group and upregulated 100 metabolites that were downregulated in the DM group, demonstrating a strong normalizing effect on the metabolic profile. Among these restored metabolites, 38 were structurally classified (Table S7).

#### Functional analysis of differential metabolites

**Figure 3.**
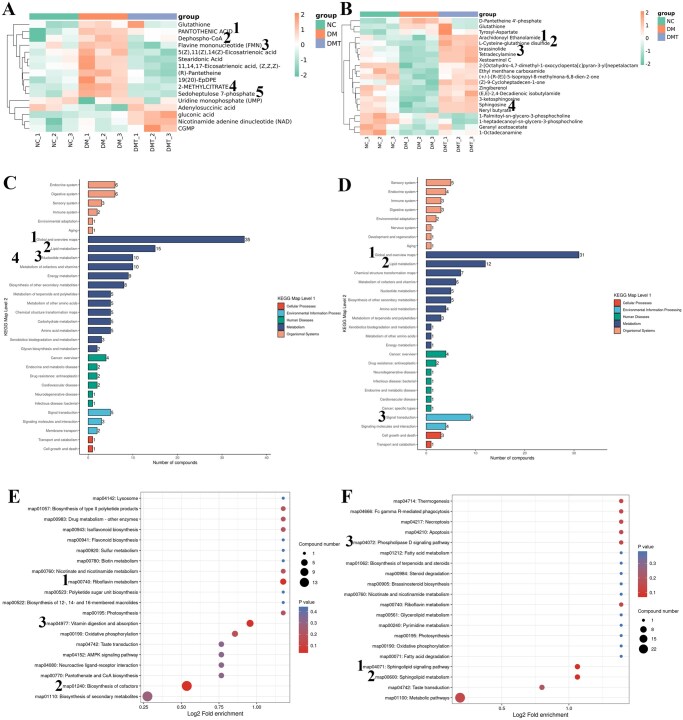
Functional analysis of significantly altered metabolites across experimental groups. (A) Hierarchical clustering of metabolites in negative mode across the three groups. Key metabolic intermediates that decreased in the DMT group compared to DM include: (1) pantothenic acid (FC = 0.58, *P *= .002), (2) dephospho-CoA (FC = 0.09, *P *= .021), (3) flavin mononucleotide (FMN; FC = 0.29, *P *= .004), (4) 2-methylcitrate (FC = 0.41, *P *= .004), and (5) sedoheptulose 7-phosphate (FC = 0.37, *P *= .001). (B) Hierarchical clustering of metabolites in positive mode across the three groups. Key anti-inflammatory metabolites increased by hUCMSC treatment include: (1) arachidonoyl ethanolamide (FC = 2.45, *P *= .001), (2) L-cysteine-glutathione disulfide (FC = 5.43, *P *= .001), (3) brassinolide (FC = 3.83, *P *= .001), and (4) sphingosine (FC = 4.44, *P *= .003). All *P*-values are Bonferroni-adjusted. KEGG pathway analysis comparing DMT and DM groups in (C) negative and (D) positive modes. Enrichment pathway analysis comparing DMT and DM groups in (E) negative and (F) positive modes. Circles represent enriched pathways, with darker shading indicating more significant metabolite variations and circle size representing pathway impact. Pathways with *P *< .05 were considered significantly enriched. Lower *P*-values indicate greater statistical significance. Number labels highlight key metabolites and pathways. NC, db/m control group; DM, untreated db/db group; DMT, hUCMSC-treated db/db group.

MetaboAnalyst 4.0 was used to generate hierarchical clustering of differential metabolites from the NC, DM, and DMT groups in negative (Figure 3A) and positive (Figure 3B) modes, respectively. Certain metabolic intermediates, such as pantothenic acid, flavin mononucleotide (FMN), dephospho-CoA, 2-methylcitrate, and sedoheptulose 7-phosphate from the redox metabolism of carbohydrates, proteins, and fats, were significantly downregulated by hUCMSCs in the DMT group compared to the DM group (*P *< .05) and were significantly elevated in the DM group compared to NC samples (*P *< .05). Conversely, the anti-inflammatory metabolites L-cysteine-glutathione disulfide, arachidonoyl ethanolamide, brassinolide, and sphingosine exhibited the opposite patterns. Tables S5-S7 provide a summary of additional data regarding the differences in metabolites between groups.

KEGG pathway analysis is a critical tool for understanding the complex interactions among metabolites. The metabolic pathways in the DM group compared to the NC group included Global and overview maps, Metabolism of cofactors and vitamins, and the Digestive system in negative mode, as well as Global and overview maps, Amino acid metabolism, and Signal transduction in positive mode, among others (Figure S4). To clarify the effects of hUCMSCs, we identified the clusters of KEGG pathways involving the differential metabolites between the DMT and DM groups (Figure 3C and D). The primary metabolic pathways identified were Global and overview maps (1), Lipid metabolism (2), Nucleotide metabolism (3), and Metabolism of cofactors and vitamins (4) in negative mode (Figure 3C) and Global and overview maps (1), Lipid metabolism (2), and Signal transduction (3) in positive mode (Figure 3D). These metabolic pathways are primarily involved in redox metabolism and signal transduction.

The primary metabolic and signal transduction pathways involved were identified through enrichment analysis based on Fisher’s exact test. MetaboAnalyst identified 47 and 31 enriched pathways between the DM and NC groups in negative and positive modes, respectively, based on rich factor and *P*-values. In negative mode (Figure S5A), three pathways exhibited the lowest *P*-values: pantothenate and CoA biosynthesis, biosynthesis of cofactors, and pentose phosphate pathway. In positive mode, the corresponding pathways were cysteine and methionine metabolism, carbapenem biosynthesis, and inflammatory mediator regulation of TRP channels (Figure S5B). Notably, arachidonoyl ethanolamide and D-pantetheine 4'-phosphate, which contribute to the latter two pathways and are strongly linked to inflammation, were restored to near-normal levels by hUCMSCs in the DMT group compared to the DM group. Between the DMT and DM groups, 44 and 46 pathways were enriched in negative and positive modes, respectively. In negative mode (Figure 3E), the top three pathways with the lowest *P*-values were riboflavin metabolism (1), biosynthesis of cofactors (2), and vitamin digestion and absorption (3); in the positive mode, the sphingolipid signaling pathway (1), sphingolipid metabolism (2), and phospholipase D signaling pathway (3) were captured, where sphingosine played a major role (Figure 3F).

### Proteomics of mouse aortic endothelial cells

#### Proteomic profiles

For single-cell type proteomic analysis, FACS was performed using a CD31 antibody to sort mouse aortic endothelial cells. Approximately 4000-4500 endothelial cells were purified from each sample (Figure S6).

**Figure 4.**
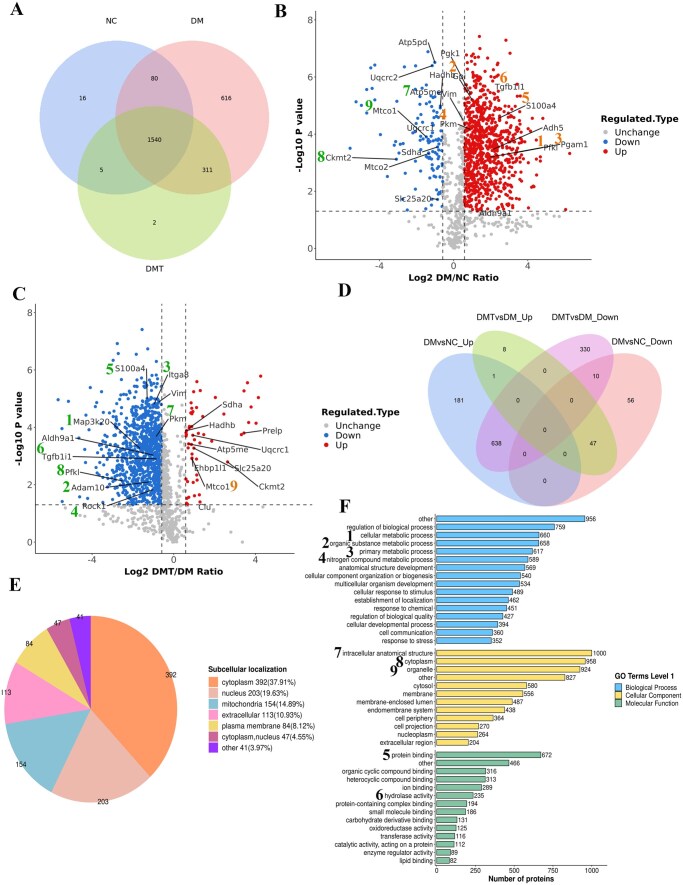
Endothelial proteomic changes in mouse aortas. (A) Venn diagram of proteins across experimental groups. (B) Volcano plot showing differentially expressed proteins between DM and NC groups: (1) Pfkl, ATP-dependent 6-phosphofructokinase (FC = 10.34, *P *< .001); (2) Gpi, glucose-6-phosphate isomerase (FC = 1.99, *P *< .001); (3) Pgam1, phosphoglycerate mutase 1 (FC = 3.98, *P *= .001); (4) Pkm, pyruvate kinase PKM (FC = 1.84, *P *< .001); (5) S100a4, protein S100-A4 (FC = 5.58, *P *< .001); (6) Tgfb1i1, transforming growth factor beta-1-induced transcript 1 protein (FC = 4.31, *P *< .001); (7) Atp5me, ATP synthase subunit e (FC = 0.43, *P *< .001); (8) Ckmt2, creatine kinase S-type (FC = 0.12, *P *= .001); (9) Mtco1, cytochrome c oxidase subunit 1 (FC = 0.36, *P *< .001). Additional proteins: Pgk1, phosphoglycerate kinase 1 (FC = 1.87, *P *< .001); Aldh9a1, 4-trimethylaminobutyraldehyde dehydrogenase (FC = 2.37, *P *= .043); Adh5, alcohol dehydrogenase class-3 (FC = 4.48, *P *< .001); Hadhb, trifunctional enzyme subunit beta (FC = 0.61, *P *< .001); Atp5pd, ATP synthase subunit d (FC = 0.50, *P *< .001); Sdha, succinate dehydrogenase (FC = 0.55, *P *< .001); Slc25a20, mitochondrial carnitine/acylcarnitine carrier protein (FC = 0.55, *P *= .019); Mtco2, cytochrome c oxidase subunit 2 (FC = 0.40, *P *< .001); Uqcrc1, cytochrome b-c1 complex subunit 1 (FC = 0.47, *P *< .001); Uqcrc2, cytochrome b-c1 complex subunit 2 (FC = 0.45, *P *< .001). (C) Volcano plot of differentially expressed proteins between DMT and DM groups: (1) Map3k20, mitogen-activated protein kinase kinase kinase 20 (FC = 0.32, *P *= .001); (2) Adam10, disintegrin and metalloproteinase domain-containing protein 10 (FC = 0.42, *P *= .008); (3) Itga8, integrin alpha-8 (FC = 0.51, *P *< .001); (4) Rock1, Rho-associated protein kinase 1 (FC = 0.49, *P *= .015); (5) S100a4 (FC = 0.41, *P *< .001); (6) Tgfb1i1 (FC = 0.55, *P *= .001); (7) Pkm (FC = 0.55, *P *< .001); (8) Pfkl (FC = 0.17, *P *= .007); (9) Mtco1 (FC = 1.85, *P *= .001). Additional proteins: Aldh9a1 (FC = 0.49, *P *= .001); Ehbp1l1, EH domain-binding protein 1-like protein 1 (FC = 1.86, *P *= .001); Prelp, prolargin (FC = 10.84, *P *< .001); Clu, clusterin (FC = 2.44, *P *= .040); Hadhb (FC = 1.54, *P *< .001); Atp5me (FC = 1.65, *P *< .001); Ckmt2 (FC = 6.22, *P *= .002); Sdha (FC = 1.69, *P *< .001); Slc25a20 (FC = 1.98, *P *= .001); Uqcrc1 (FC = 1.91, *P *< .001). All *P*-values are Bonferroni-adjusted. (D) Effects of hUCMSC treatment on endothelial proteins altered in diabetic mice. (E) Subcellular localization of differentially expressed proteins between DMT and DM groups. (F) Gene Ontology (GO) enrichment analysis of altered proteins between DMT and DM groups. Number labels indicate key proteins and pathways. DM, untreated db/db group; DMT, hUCMSC-treated db/db group; NC, db/m control group.

A total of 605 705 MS/MS spectra were matched to 18 358 peptides in the NC, DM, and DMT groups, which corresponded to 2570 distinct and 1780 quantified proteins. **Figure 4A** shows the Venn diagram of the proteins in the various groups.

A total of 2568 proteins were identified in the NC and DM groups. Using a fold-change threshold of >1.50 or <0.67 with *P *< .05, we identified 933 proteins with significantly altered abundance, of which 820 were upregulated and 113 were downregulated in the DM group compared to the NC group. Several glycolytic enzymes were upregulated, including ATP-dependent 6-phosphofructokinase (Pfkl, Pfkm, Pfkp), glucose-6-phosphate isomerase (Gpi), phosphoglycerate kinase 1 (Pgk1), phosphoglycerate mutase 1 (Pgam1), and pyruvate kinase PKM (Pkm), consistent with the metabolomics results. Fatty acid metabolism-related enzymes, such as 4-trimethylaminobutyraldehyde dehydrogenase (Aldh9a1) and alcohol dehydrogenase class-3 (Adh5), were also elevated. In contrast, proteins associated with mitochondrial function were decreased, including ATP synthase subunits (Atp5me, Atp5pd), creatine kinase S-type (Ckmt2), succinate dehydrogenase flavoprotein subunit (Sdha), mitochondrial carnitine/acylcarnitine carrier protein (Slc25a20), and cytochrome c oxidase subunits (Mtco1, Mtco2, Cox5a, Cox5b, Cox6c, Cox7a1, Cox7c, Uqcrq, Uqcrfs1, Uqcrb). Consistent with morphological observations, the DM group exhibited higher levels of EndMT markers, such as Protein S100-A4 (S100a4), Transforming Growth Factor beta-1-induced transcript 1 protein (Tgfb1i1/Hic-5), and Calponin 2 (Cnn2), as well as proteins involved in transcriptional and translational activity, including Elongin-B (Elob), Elongin-C (Eloc), and 40S ribosomal protein S6 (Rps6), compared to the NC group (Figure 4B).

Of the 933 proteins altered in the DM group compared to the NC group, 685 were restored to near-normal levels in the DMT group relative to the DM group. This included 47 upregulated and 638 downregulated proteins (Figure 4C and D). These 685 differentially expressed proteins were associated with critical biological processes including inflammation, metabolism, mitochondrial function, and EndMT. hUCMSC treatment downregulated detrimental proteins in the DMT group compared to the DM group, as confirmed by endothelial co-staining. These included Rho-associated protein kinase 1 (Rock1), implicated in cell transformation and migration; Disintegrin and metalloproteinase domain-containing protein 10 (Adam10), involved in inflammation; and integrin alpha-8 (Itga8), associated with mesenchymal regulation. Conversely, hUCMSCs upregulated proteins supporting endothelial function, including Clusterin (Clu), Prolargin (Prelp), and EH domain-binding protein 1-like protein 1 (Ehbp1l1) (Table S8).

#### Functional analysis of aortic endothelial proteomics regulated by hUCMSCs

Subcellular localization analysis of differentially expressed proteins in aortic endothelial cells following hUCMSC treatment was performed using CELLO (version 2.5) for the DMT group compared to the DM group (Figure 4E). Of the 1034 localized proteins, 392 (37.91%) were cytoplasmic, 203 (19.63%) nuclear, 154 (14.89%) mitochondrial, and 113 (10.93%) extracellular.

GO enrichment analysis categorized proteins in the DMT group compared to the DM group into three main series: cellular component, molecular function, and biological process. Within the biological process category, most proteins participated in metabolic processes, including cellular (1), organic substance (2), primary (3), and nitrogen compound metabolic processes (4). In the molecular function classification, the majority of proteins were associated with binding (5) and hydrolase activity (6). For the cellular component, proteins were primarily localized to the intracellular anatomical structure (7), cytoplasm (8), and organelles (9) (Figure 4F).

**Figure 5.**
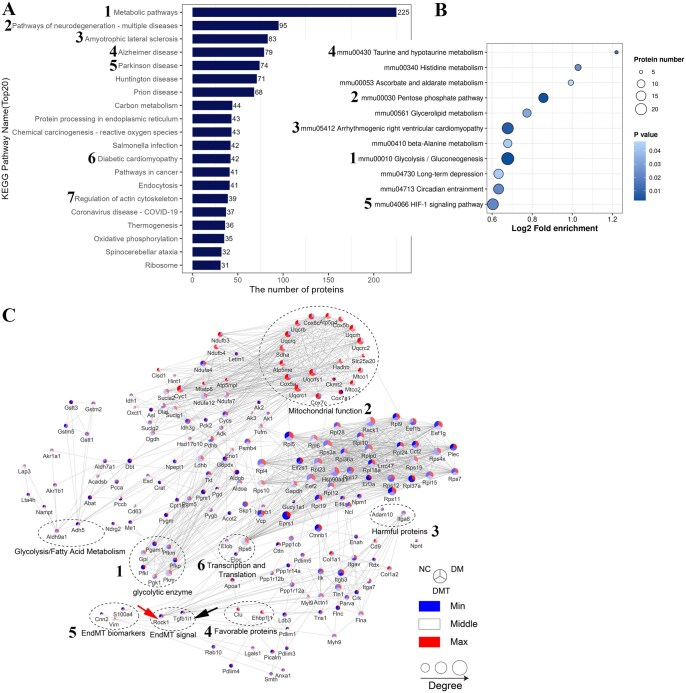
Further functional analysis of endothelial proteomics in mouse aortas. (A) Top 20 KEGG pathways modulated by hUCMSCs based on endothelial proteomics. (B) Enriched pathways regulated by hUCMSCs based on endothelial proteomics. (C) Protein–protein interaction network analysis of differentially expressed proteins among NC, DM, and DMT groups. Functional categories include: (1) Glycolysis and fatty acid metabolism: Pfkl, Pfkm, Pfkp, Gpi, Pgk1, Pgam1, Pkm, Aldh9a1, Adh5; (2) Mitochondrial function: Ckmt2, Sdha, Cox5a, Cox5b, Cox6c, Cox7a1, Cox7c, Uqcrc1, Uqcrc2, Uqcrq, Uqcrfs1, Hadhb, Uqcrb, Atp5me, Atp5pd, Slc25a20, Mtco1, Mtco2, Uqcrh; (3) Proteins detrimental to endothelial function: Adam10, Itga8; (4) Proteins beneficial to endothelial function: Ehbp1l1, Clu; (5) EndMT-related proteins: S100a4, Cnn2, Tgfb1i1, Rock1; (6) Transcription and translation: Elob, Eloc, Rps6. The black arrow indicates Tgfb1i1; the red arrow indicates Rock1. Number labels highlight key proteins and pathways. DM, untreated db/db group; DMT, hUCMSC-treated db/db group; NC, db/m control group.

The top 20 KEGG pathways in the DMT group compared to the DM group are shown in Figure 5A. Metabolic pathways contained the highest number of proteins (1. 225), followed by neurodegeneration-multiple diseases pathways (2. 95), amyotrophic lateral sclerosis (3. 83), Alzheimer’s disease (4. 79), and Parkinson’s disease (5. 74). Interestingly, proteins associated with nervous system diseases were primarily linked to microtubule function, cytoplasmic dynein binding to organelles, and synapse formation during brain or cell development, suggesting a role in cytoskeletal organization. Pathways related to diabetic cardiomyopathy (6. 42 proteins) and regulation of the actin cytoskeleton (7. 39 proteins) were more closely associated with cellular fibrosis and mesenchymal characteristics. Additionally, the TGF-beta signaling pathway (6 proteins), Ras signaling pathway (14 proteins), and MAPK signaling pathway (17 proteins) were annotated as transducing signals for EndMT and TGF-family members, particularly highlighting Rock1 in the TGF-beta signaling pathway. KEGG pathway enrichment analysis of differentially expressed proteins was performed using Fisher’s exact test. The pathways most significantly regulated by hUCMSCs included Glycolysis/Gluconeogenesis (1), Pentose phosphate pathway (2), Arrhythmogenic right ventricular cardiomyopathy (3), Taurine and hypotaurine metabolism (4), and the HIF-1 signaling pathway (5) (Figure 5B).

#### Protein–protein interactions network analysis

Protein–protein interactions network analysis of the differential proteins was performed to characterize functional interactions among proteins in the NC, DM, and DMT groups. Altered proteins clustered into five major categories. The primary cluster consisted of metabolic proteins involved in glycolysis and fatty acid metabolism, such as Pkm, Pfkl, Pfkm, Pfkp, and Adh5, which were elevated in the DM group compared to the NC group and decreased after hUCMSC treatment. The second cluster, which included mitochondrial proteins such as Slc25a20, Mtco1, Mtco2, and Uqcrc1, exhibited an inverse expression pattern. The third cluster, associated with inflammation, adhesion, and migration, was subdivided into two groups: detrimental proteins that impair vascular endothelium (e.g., Adam10 and Itga8) were upregulated in the DM group and downregulated by hUCMSCs, whereas beneficial proteins that support endothelial function (e.g., Ehbp1l1 and Clu) showed the opposite trend. Notably, these pro-inflammatory proteins consistently co-varied with EndMT-related signaling proteins such as Tgfb1i1 and Rock1. The fourth cluster included spliceosomal, ribosomal, and proteasomal components such as Elob, Eloc, and Rps6, which are involved in transcription, translation, and protein homeostasis. These were generally upregulated in the DM group and downregulated by hUCMSCs. Finally, hUCMSCs downregulated the fifth cluster of EndMT markers, including S100a4 and Cnn2, which were upregulated in the DM group. These results suggest that hUCMSCs ameliorate inflammation-induced EndMT and vascular remodeling in diabetic mice, likely through the Tgfb1i1/Rock1 pathway (Figure 5C and Table S8).

### Comprehensive analysis of metabolomics and proteomics

**Figure 6.**
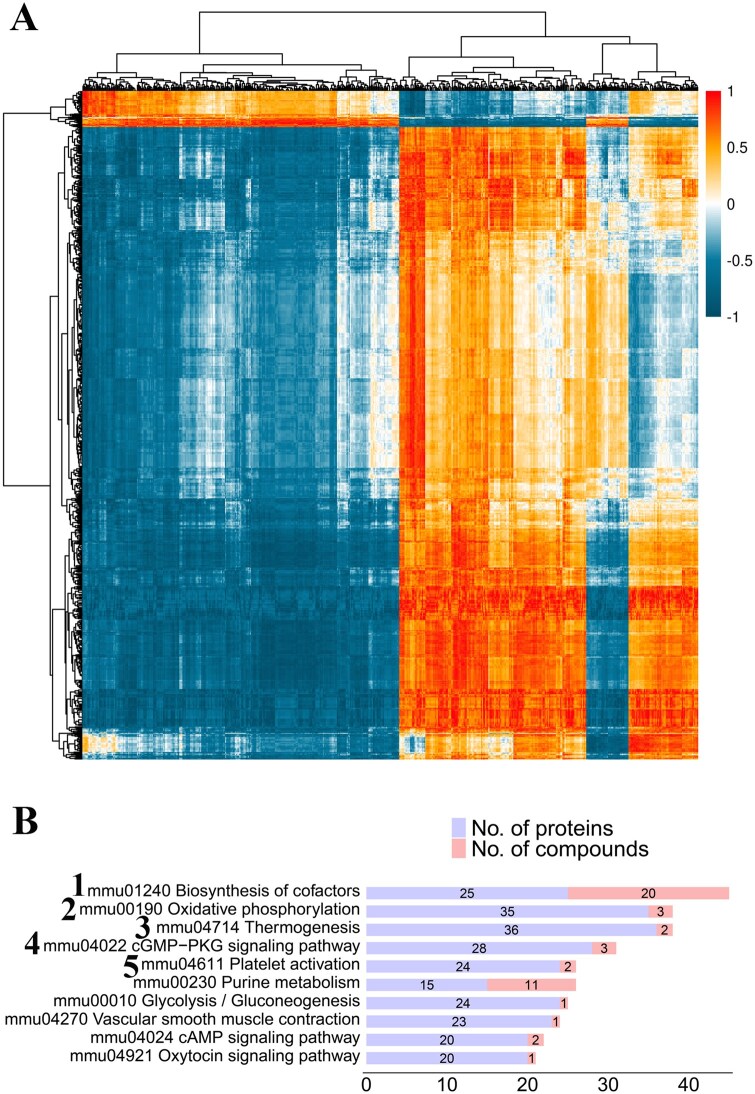
Integrated analysis of metabolomics and endothelial proteomics. (A) Correlation clustering heatmap from integrated analysis of metabolomics and endothelial proteomics between DMT and DM groups. Color indicates correlation coefficient (*r*), with red representing *r* > 0 and blue representing *r* < 0. Differential proteins are shown on the horizontal axis and differential metabolites on the vertical axis. (B) Top 10 shared pathways from integrated analysis of metabolomics and endothelial proteomics between DMT and DM groups. Number labels highlight key pathways. NC, db/m control group; DM, untreated db/db group; DMT, hUCMSC-treated db/db group.

Differentially abundant proteins and metabolites in the DMT group showed significant correlations compared to the DM group (Figure 6A). The top five pathways shared by these molecules were: biosynthesis of cofactors (1), oxidative phosphorylation (2), thermogenesis (3), cGMP-PKG signaling pathway (4), and platelet activation (5) (Figure 6B). The first three pathways were associated with general metabolic processes; the cGMP-PKG pathway was closely linked to nitric oxide (NO) signaling and endothelial function; and proteins involved in platelet activation primarily contributed to cytoskeletal organization and inflammatory response. Combined with morphological characteristics, these findings demonstrate that hUCMSCs improve aortic endothelial function, thereby alleviating vascular remodeling and attenuating the atherosclerotic trend in diabetic mice.

### Target molecules confirmed by endothelial co-staining and RT-PCR *in vitro*

**Figure 7.**
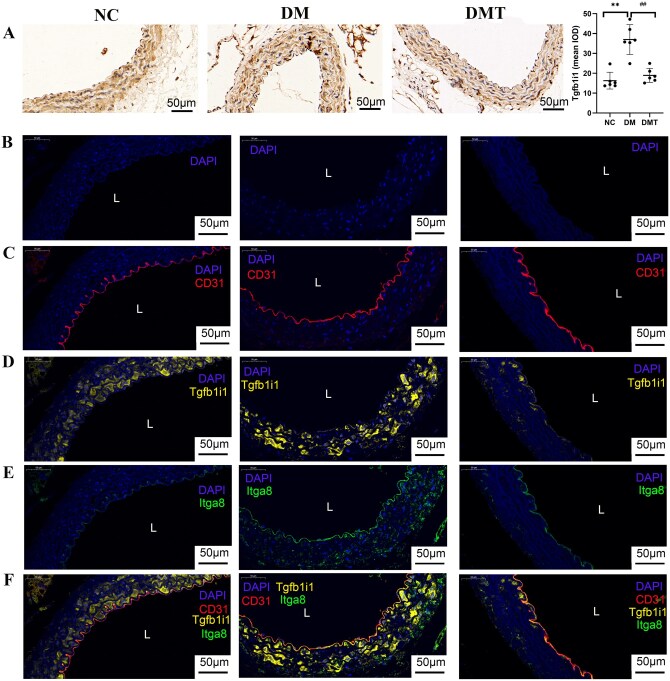
Verification of targeted endothelial proteins (×400). (A) Immunohistochemical staining of transforming growth factor beta-1-induced transcript 1 protein (Tgfb1i1). (B-F) Co-staining of Tgfb1i1 (yellow), Itga8 (green), CD31 (red), and nuclei (blue). (B) Nuclei (blue), (C) CD31 (red) and nuclei (blue), (D) Tgfb1i1 (yellow) and nuclei (blue), (E) Itga8 (green) and nuclei (blue), (F) Merged image of Tgfb1i1 (yellow), Itga8 (green), CD31 (red), and nuclei (blue). All scale bars = 50 μm. IOD, integrated optical density; L, lumen. NC, db/m control group; DM, untreated db/db group; DMT, hUCMSC-treated db/db group. **P < .01 vs NC group; ##*P *< .01 vs DM group (Bonferroni-adjusted).

**Figure 8.**
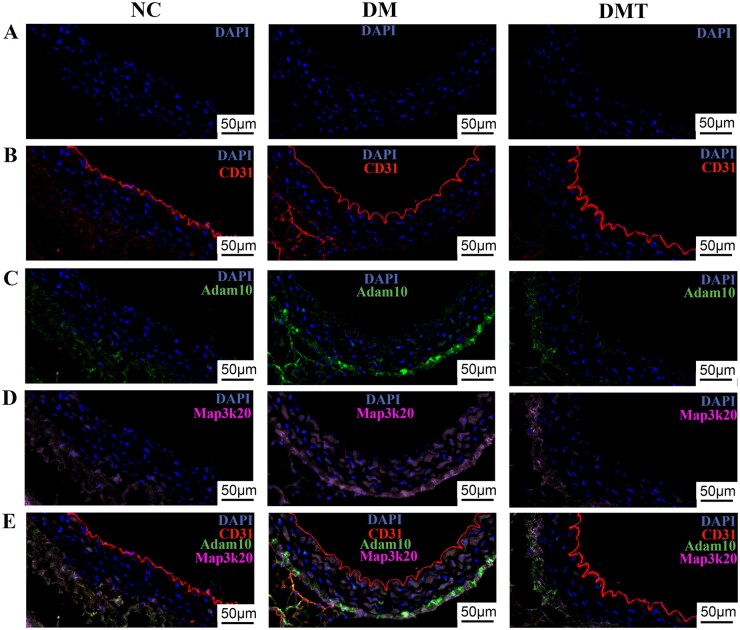
Co-staining of inflammatory molecules adam10 and map3k20 with cd31 and nuclei. (A) Nuclei (blue). (B) Co-staining of CD31 (red) and nuclei (blue). (C) Co-staining of Adam10 (green) and nuclei (blue). (D) Co-staining of Map3k20 (pink) and nuclei (blue). (E) Merged image of Adam10 (green), Map3k20 (pink), CD31 (red), and nuclei (blue). Adam10, disintegrin and metalloproteinase domain-containing protein 10; Map3k20, mitogen-activated protein kinase kinase kinase 20. All scale bars = 50 μm. DM, untreated db/db group; DMT, hUCMSC-treated db/db group; NC, db/m control group.

**Figure 9.**
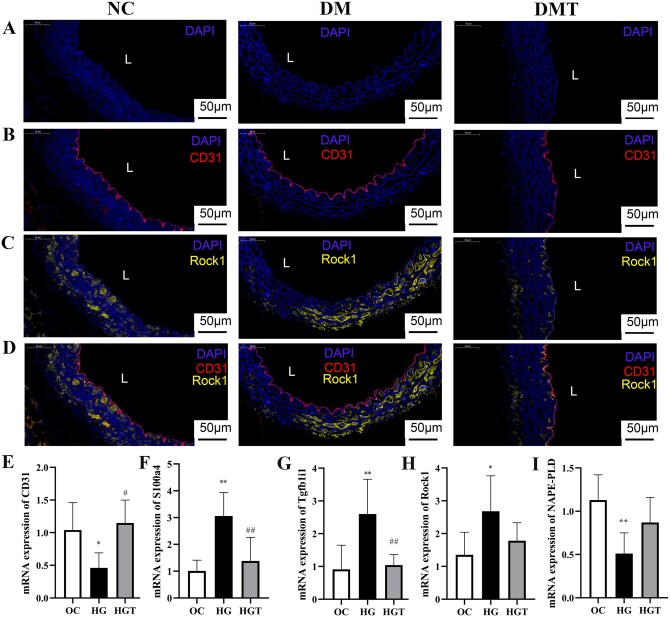
Rock1 Verification by endothelial co-staining *in vivo* and RT-PCR *in vitro*. (A-D) Co-staining of signaling molecule Rock1 with CD31: (A) nuclei (blue), (B) CD31 (red) and nuclei (blue), (C) Rock1 (yellow) and nuclei (blue), (D) merged image of Rock1 (yellow), CD31 (red), and nuclei (blue). All scale bars = 50 μm. L, lumen. NC, db/m control group; DM, untreated db/db group; DMT, hUCMSC-treated db/db group. (E-I) Rock1 inhibition by fasudil alleviates high glucose-induced EndMT *in vitro* but does not significantly affect the principal AEA synthase NAPE-PLD: relative mRNA expression of (E) CD31, (F) S100a4, (G) Tgfb1i1, (H) Rock1, and (I) NAPE-PLD in endothelial cells. AEA, arachidonoyl ethanolamide; HG, high glucose (30 mM glucose); HGT, high glucose treatment group (30 mM glucose + 10 μM fasudil); NAPE-PLD, N-acyl phosphatidylethanolamine-hydrolyzing phospholipase D. OC, osmotic control (5.6 mM glucose + 24.5 mM mannitol). *P < .05, **P < .01 vs OC group; #*P *< .05, ##*P *< .01 vs HG group (Bonferroni-adjusted).

Immunohistochemical staining confirmed that Tgfb1i1, a key EndMT-related protein, was elevated in the DM group compared to the NC group and reduced by hUCMSC treatment in the DMT group (Figure 7A). Co-staining of the adhesion and inflammation-related molecules Itga8, Adam10, and Map3k20 with CD31 showed their expression in the aortic endothelium was upregulated in the DM group and downregulated after hUCMSC treatment in the DMT group (Figures 7B and 8). Furthermore, endothelial co-staining verified that Rock1, a key signaling molecule in the Tgfb1i1/Rock1 pathway identified by KEGG analysis, was more strongly expressed in the DM group and attenuated in the DMT group (Figure 9A-D). To further examine the role of high glucose and the Tgfb1i1/Rock1 pathway in EndMT, endothelial mRNA expression was analyzed *in vitro*. Levels of Tgfb1i1, Rock1, and the EndMT marker S100a4 were increased under high glucose (30 mM) exposure, while the endothelial marker CD31 and NAPE-PLD (N-acyl phosphatidylethanolamine-hydrolyzing phospholipase D), the primary synthase of the anti-inflammatory metabolite arachidonoyl ethanolamide, were decreased (Figure 9E-I). Fasudil, a Rock1 inhibitor, reversed the changes in S100a4, CD31, and Tgfb1i1 expression (Figure 9E-H). These results are consistent with endothelial cell-type proteomics and integrated metabolomic-proteomic analyses.

## Discussion

In this study, a lower, repeated-dose regimen was employed instead of a single large bolus, better recapitulating the potential continuous, low-level engraftment of stem cells in diabetic patients. This dosing strategy was designed to promote a more stable and protective microenvironment, thereby enhancing the robustness and clinical relevance of the treatment effect. This approach has proven valuable for evaluating the efficacy in diabetic complications, as demonstrated in previous studies.^12^ A modest and transient reduction in fasting blood glucose was observed at one time point; however, hUCMSC treatment did not achieve sustained glycemic control, as indicated by unchanged GSP levels and body weight. Meanwhile, hUCMSC administration partially restored the anti-inflammatory cytokine IL-10 but did not significantly alter levels of the key pro-inflammatory cytokine IL-6, suggesting a selective immunomodulatory effect. While previous reports have described hypoglycemic^13^ and anti-inflammatory^14^ properties of MSCs, we speculate that factors such as disease model severity, dosage, or treatment timing may underlie the modest efficacy observed here. Subsequently, our results demonstrated that hUCMSCs alleviated aortic remodeling, accompanied by reduced collagen deposition, decreased expression of the endothelial marker CD31, and increased levels of the mesenchymal markers Transgelin and S100A4, all established features of EndMT.^15^ These findings were further supported by aortic ultrastructural analysis using TEM. The amelioration of vascular pathology may be attributed to specific biological activities of hUCMSCs, as revealed by metabolomic and endothelial proteomic analyses, rather than a comprehensive reversal of hyperglycemia or systemic inflammation.

Metabolomic analysis revealed that hUCMSCs downregulated most of the elevated intermediate metabolites of carbohydrate metabolism in diabetes, such as 2-methylcitrate, dephospho-CoA, and D-pantetheine 4'-phosphate. hUCMSC treatment also improved the enrichment patterns of central carbon metabolism and energy metabolism pathways in diabetic mice. Metabolic reprogramming and the inflammatory microenvironment are recognized as central mediators of diabetic complications.^16^^,^^17^ Furthermore, hUCMSCs upregulated the levels of anti-inflammatory metabolites that were reduced in diabetes, including sphingosine and arachidonoyl ethanolamide. Arachidonoyl ethanolamide (AEA), also known as anandamide, is a lipid messenger with diverse roles in health and disease. It acts as a potent anti-inflammatory mediator in endothelial cells and is involved in energy metabolism, anxiety, and inflammation.^18^^,^^19^ We also observed that NAPE-PLD, the primary enzyme responsible for AEA synthesis, was decreased in endothelial cells exposed to high glucose *in vitro*. Previous studies have shown that NAPE-PLD enhances efferocytosis by bone marrow-derived macrophages to ameliorate cardiometabolic diseases.^20^ However, in our study, Rock1 inhibition by fasudil did not significantly affect NAPE-PLD levels. These results suggest that downregulation of AEA and NAPE-PLD contributes to the diabetic microenvironment and subsequent EndMT, and that hUCMSCs regulate this process through mechanisms independent of Rock1 signaling. Sphingolipids, common components of cell membranes, are increasingly recognized as critical regulators of multiple signaling pathways, particularly those maintaining vascular endothelial barrier integrity. Their antagonism against TGF-β and other pro-fibrotic pathways has also made them a subject of interest in fibrosis research.^21^ Collectively, our findings demonstrate that hUCMSCs ameliorate metabolic reprogramming and subsequent inflammatory and fibrotic alterations in the vascular microenvironment of diabetic mice.

ECs are highly heterogeneous and plastic, and the metabolic reprogramming and inflammatory microenvironment can reprogram them from atheroprotective to proatherogenic phenotypes, including EndMT.^22^^,^^23^ Biological changes can be directly linked to a particular cell type in single-cell type analysis rather than being averaged across complex tissues or ensembles.^24^^,^^25^ In our study, endothelial cell type proteomics found that Pfkl, Pfkm and Pfkp involved in the first step of glycolysis, Gpi in the second step, Pgam1 in the crucial step, Pkm in the final rate-limiting step, and Pgk1 catalyzing ATP production in glycolysis, were increased in aortic endothelia of diabetic mice and reversed by hUCMSCs, as well as Aldh9a1 and Adh5 in fatty acid metabolism.^26^ On the other hand, Hadhb and Slc25a20 in mitochondrial β-oxidation, Atp5me and Atp5pd as mitochondrial membrane ATP synthases, Ckmt2 in energy transduction, Sdha cytochrome b-c1 complex, and Cytochrome c oxidases in the mitochondrial electron transport chains were impaired in diabetic aortic endothelia and restored by hUCMSCs. Our findings established a molecular mechanism underlying the protection of hUCMSCs in metabolic reprogramming and vascular microenvironment. Interestingly, the levels of S100a4 (also known as fibroblast-specific protein-1, FSP-1) and Cnn2, which are recognized as mesenchymal markers,^27^ were increased in the aortic endothelium of diabetic mice, indicating EndMT progression, and were downregulated by hUCMSCs. EndMT is driven by transcription factors that alter gene expression, leading to a transition from an endothelial to a mesenchymal phenotype in both morphology and physiology.^28^ Our study demonstrated that hUCMSCs consistently affected proteins involved in gene expression, such as Elob, Eloc,^29^ and Rps6,^30^ along with mesenchymal markers. In the aortic endothelium of diabetic mice, hUCMSCs modulated the turnover of proteins that help maintain the endothelial phenotype. These proteins included Ehbp1l1, which regulates apical-directed transport in polarized epithelial cells;^31^ Prelp, which functions as a molecular anchor between basement membrane constituents and the extracellular matrix (ECM);^32^ and Clu, which promotes cellular survival through cytoprotection.^33^ Moreover, previous reports indicate that TGF-β signaling, a master regulator of EndMT, is inhibited by both Prelp and Clu.^4^ These findings support the efficacy of hUCMSCs and highlight the potential of developing strategies to prevent EndMT and subsequent aortic remodeling in diabetic patients.

We next investigated the molecular mechanisms underlying hUCMSC-mediated protection and identified several factors and signaling pathways involved in EndMT. Our findings showed that aortic endothelial cells from diabetic mice exhibited elevated levels of Tgfb1i1, Rock1, Itga8, Map3k20, and Adam10, all closely associated with EndMT, which were largely restored by hUCMSC treatment. Tgfb1i1, also known as hydrogen peroxide-inducible clone-5 (Hic-5), functions as a cofactor for cellular TGF-β1 and interacts with various nuclear receptors. Previous studies have shown that Tgfb1i1 promotes focal adhesion formation, regulates actin cytoskeleton and vimentin organization, and facilitates epithelial-to-mesenchymal transition (EMT), likely through canonical TGF-β signaling and the RhoA/ROCK pathway.^34^^,^^35^ As a downstream effector of TGF-β, Rock1 has been shown to induce EndMT in diabetic nephropathy by regulating cytoskeletal remodeling, mitochondrial fission, and apoptosis.^36^^,^^37^ In our study, Rock1 exhibited expression trends similar to Tgfb1i1, and functional analysis clustered Tgfb1i1/Rock1 together as central to the EndMT process. We found that fasudil, a Rock1 inhibitor, alleviated high glucose-induced EndMT *in vitro*. Previous reports indicate that fasudil also downregulates EndMT and ameliorates Fabry disease phenotypes, including left ventricular hypertrophy and renal fibrosis.^38^ Consistently, multipotent mesenchymal stem cells (MMSCs) have been shown to reduce Rock1/2 expression, thereby inhibiting neuroinflammatory and apoptotic pathways and providing a sustainable therapeutic strategy for vascular dementia.^39^ Similarly, inhibition of ROCK1/2 activity in type 2 diabetes-induced pluripotent stem cell-derived hepatocytes restored aspects of insulin signaling.^40^ Our study extends the current understanding by demonstrating that hUCMSCs attenuate diabetes-induced EndMT through suppression of the Tgfb1i1/ROCK1 pathway. Furthermore, Itga8 expression was shown to be elevated in liver fibrosis specimens and has been identified as a specific cell surface marker of perivascular mesenchymal cells in the developing liver.^41^ Our findings further suggest that Itga8 may be an important molecule in Tgfb1i1/Rock1 activation and EndMT progression. Chronic inflammation has been demonstrated to significantly influence EndMT, vascular remodeling, and atherosclerosis.^42^ Map3k20, a member of the MAPKKK signal transduction family, forms homodimers that regulate inflammatory cytokines and the JNK/MAPK pathway.^43^ Previous studies have shown that Adam10 plays multiple roles by shedding or modifying proteins, thereby controlling inflammatory responses, tissue remodeling, developmental processes, and proliferative signaling pathways.^44^ Although MSCs have demonstrated considerable therapeutic potential for conditions including diabetes and hematological malignancies, their clinical application faces challenges related to intrinsic differences among cell sources and nonstandardized production methods.^45^^,^^46^ For example, while MSC co-infusion can reduce graft-versus-host disease (GVHD) incidence, it may also increase the risk of disease relapse.^47^ To address these limitations, advanced culture systems such as microcarrier-based platforms and optimized operational strategies have been developed.^48^ The immune-privileged properties of MSCs are highly dependent on the microenvironment, leading to variable functional outcomes. Notably, hUCMSCs have demonstrated superior efficacy in improving hyperglycemia and metabolic dysfunction-associated fatty liver disease.^49^ Accordingly, this study employed a lower, repeated-dose regimen of hUCMSCs. Collectively, our findings reveal a novel molecular mechanism through which hUCMSCs may reduce the inflammatory microenvironment via interactions with Tgfb1i1 and Rock1, thereby mitigating EndMT progression and aortic remodeling in diabetes.

Our study has several limitations. First, the sample size, while consistent with other preclinical studies in this field, remains relatively small. Although it was adequate to detect the large effect sizes observed in our primary outcomes, the limited sample size may affect the generalizability of our findings and the statistical power to identify more subtle effects. Future studies with larger cohorts will be valuable to confirm and extend these observations. Second, although our endothelial proteomic analysis identified numerous differentially expressed proteins, the functional roles of most candidates remain unvalidated in this study. This represents a common but important constraint of high-throughput discovery approaches. We have proposed specific mechanisms and hypotheses for the top candidate proteins, Tgfb1i1 and Rock1, and have performed preliminary validation in endothelial cells. Future work should include functional gain-of-function and loss-of-function experiments, such as gene knockdown or overexpression in *in vitro* and *in vivo* models, to definitively establish their causal roles in the phenotypic effects mediated by hUCMSCs. Third, our investigation was conducted in a rodent model of diabetes, and known discrepancies exist between animal models and human pathophysiology. The therapeutic potential of these identified targets will require validation in human-specific models or future clinical studies.

## Conclusion

In summary, current proteomic approaches generally operate beyond single-cell resolution. This study sought to address this limitation by using fluorescence-activated cell sorting (FACS) to purify aortic endothelial cells from db/db mice, integrated with metabolomic profiling. This strategy enabled a preliminary mapping of metabolic reprogramming in the vascular microenvironment and protein regulatory networks within the endothelial organization during diabetic aortic remodeling. Through this approach, we uncovered a previously unrecognized role of hUCMSCs in modulating metabolic reprogramming and EndMT and identified the Tgfb1i1/Rock1 axis as a key regulator driving the loss of endothelial characteristics in diabetes. From a clinical and translational perspective, this work provides a methodology to extract molecular insights embedded in tissue samples for diagnostic and prognostic purposes, and to explore new therapeutic strategies for diabetic aortic remodeling.

## Supplementary Material

szaf077_Supplementary_Data

## Data Availability

The datasets presented in this study are available in online repositories. The repository names and accession number(s) can be found at: ProteomeXchange, PXD057091, http://proteomecentral.proteomexchange.org/cgi/GetDataset? ID=PXD057091.
