# Supplementary material for: The protection of mesenchymal stem cells in metabolic reprogramming and endothelial-mesenchymal transition in diabetic aortas
Source: Stem Cells Transl Med. 2026 Jan 13;15(1):szaf077. doi: 10.1093/stcltm/szaf077 (PMC12803787; doi:10.1093/stcltm/szaf077)
Supplement: szaf077_Supplementary_Data [file szaf077_supplementary_data.zip › Supplementary Information-SCTM-25-0021.pdf]

Supplementary Information for:

The protection of mesenchymal stem cells in metabolic reprogramming and endothelial-mesenchymal transition in diabetic aortas

Mingying Ling[1], Jingxian He[1,2], Xu Jia[1], Na Yu[3,4], Yiping Song[1], Xuehui Li[1], Congmin Tang[1,2], Wenzhuo Yu[1,2], Han Qiao[1,2], Chenglong Zhang[1,2], Zhen Zhang[1], Tianmin Ma[5], Chuanli Zhao[1], Yanqiu Xing[1]*

[1]Department of Geriatric Medicine, Laboratory of Gerontology and Anti-aging Research, Jinan Clinical Research Center for Geriatric Medicine, Qilu Hospital of Shandong University, 250012 Jinan, Shandong, China

[2]Institute of Basic Medical Sciences, Qilu Hospital of Shandong University, 250012 Jinan, Shandong, China

[3]Shandong Precision Medicine Engineering Laboratory of Bacterial Anti-tumor Drugs, 250101 Jinan, Shandong, China

[4]College of Clinical Medicine, Shandong University, 250012 Jinan, Shandong, China

[5]Service Improvement and Innovation, Te Whatu Ora Health New Zealand, 15 Shea Terrace, Auckland 2014, New Zealand

***Corresponding author:** Yanqiu Xing, MD, PhD, Department of Geriatric Medicine, Laboratory of Gerontology and Anti-Aging Research, Qilu Hospital of Shandong University, 250012 Jinan, Shandong Province, China.
E-*mail address:* xingyanqiu@sina.com.

This file contains supplementary Figures S1-S6, Tables S1-S8, and additional experimental details.

Supplementary Figures

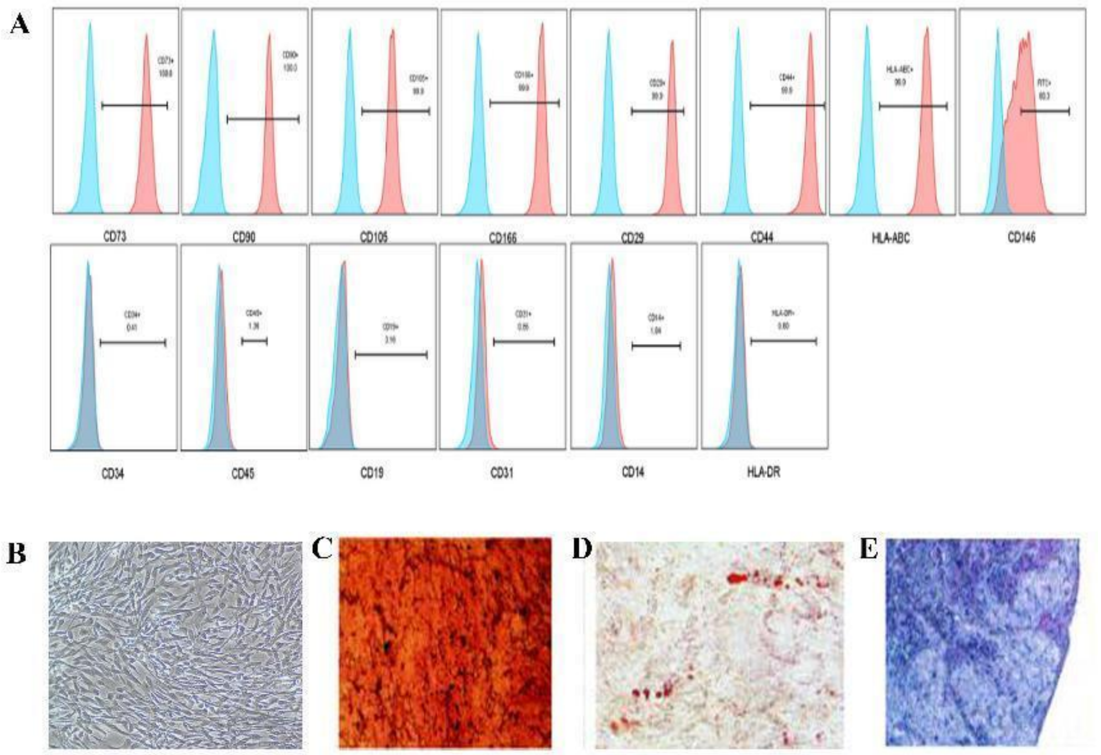

Supplementary Fig. 1. Confirmation of human umbilical cord mesenchymal stem cells (hUCMSCs). **(A)** Flow cytometric examination of cell surface markers of MSCs. **(B)** The cell morphology of hUCMSCs (passage 5) was showed under a light microscope (magnification×100). **(C-E)** Representative images of osteocyte (×100), adipocyte (×400), and chondrocyte (×200) differentiation of hUCMSCs cultured in the differentiation media. The cells were examined by cytochemical staining with Alizarin Red **(C)**, Oil red O **(D)**, and Alcian Blue **(E)**, respectively.

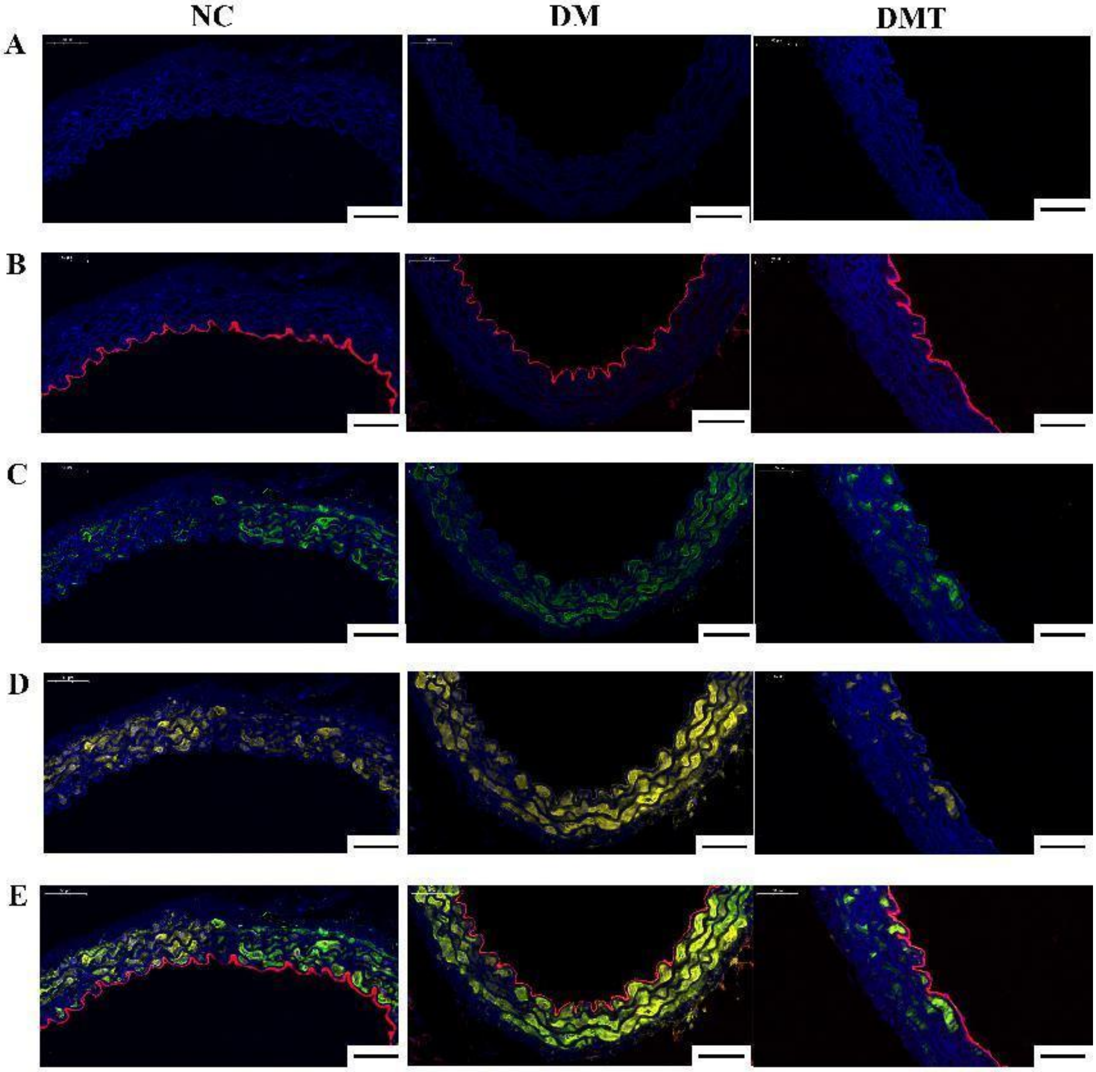

Supplementary Fig. 2. Co-staing of EndMT markers Transgelin (green), S100A4 (yellow) with CD31 (red) and nuclei (blue). **(A)** nuclei (blue). **(B)** Co-staing of CD31 (red) and nuclei (blue). **(C)** Co-staing of Transgelin (green) and nuclei (blue). **(D)** Co-staing of S100A4 (yellow) and nuclei (blue). **(E)** Merged image of Transgelin (green), S100A4 (yellow) with CD31 (red) and nuclei (blue). EndMT, endothelial-to-mesenchymal transition. All scale bars=50μm. NC, control db/m group; DM, untreated db/db group; DMT, human umbilical cord mesenchymal stem cells (hUCMSCs) treated db/db group.

A

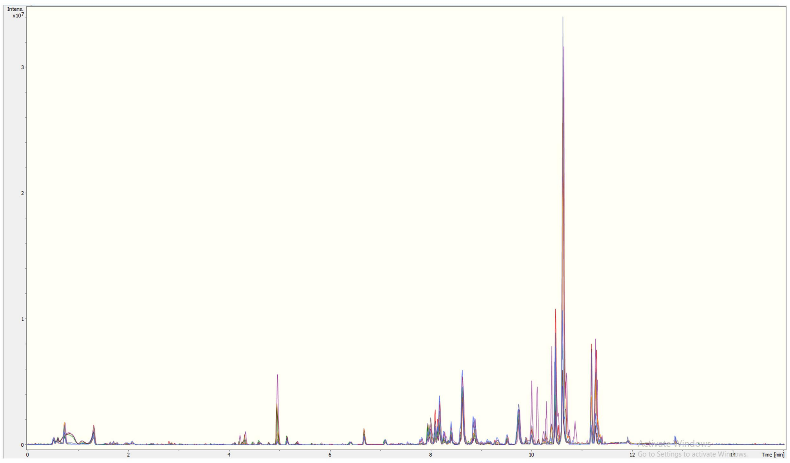

B

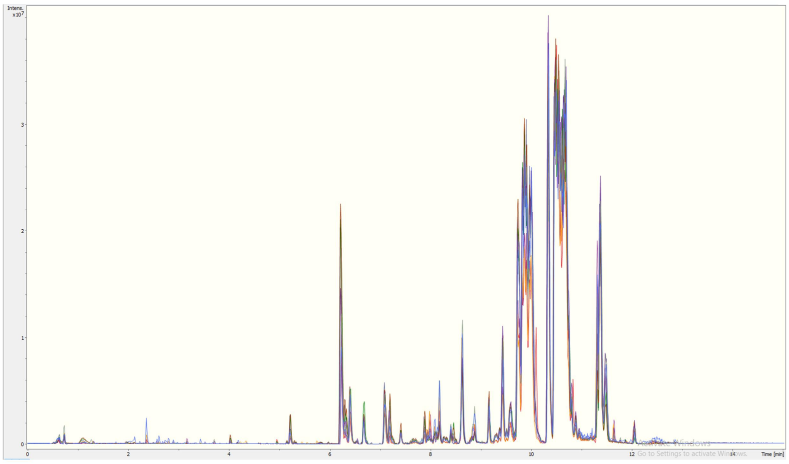

Supplementary Fig. 3. Representative (liquid chromatography-mass spectrometry) LC-MS negative **(A)** and positive **(B)** total ions of current chromatograms (TIC) of aortas in mice.

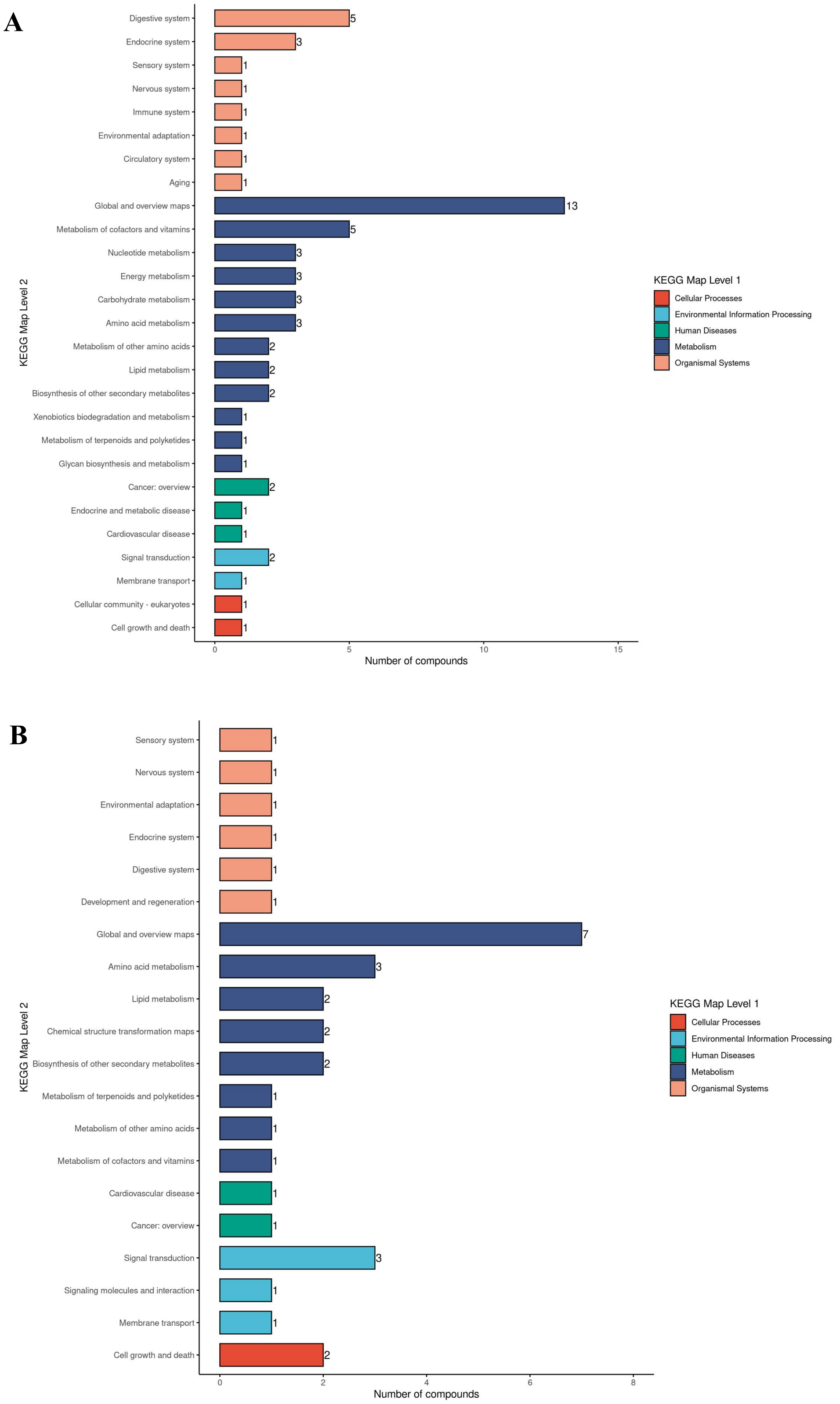

Supplementary Fig. 4. KEGG pathway analysis between DM and NC groups in negative **(A)** and positive mode **(B)**. NC, control db/m group; DM, untreated db/db group.

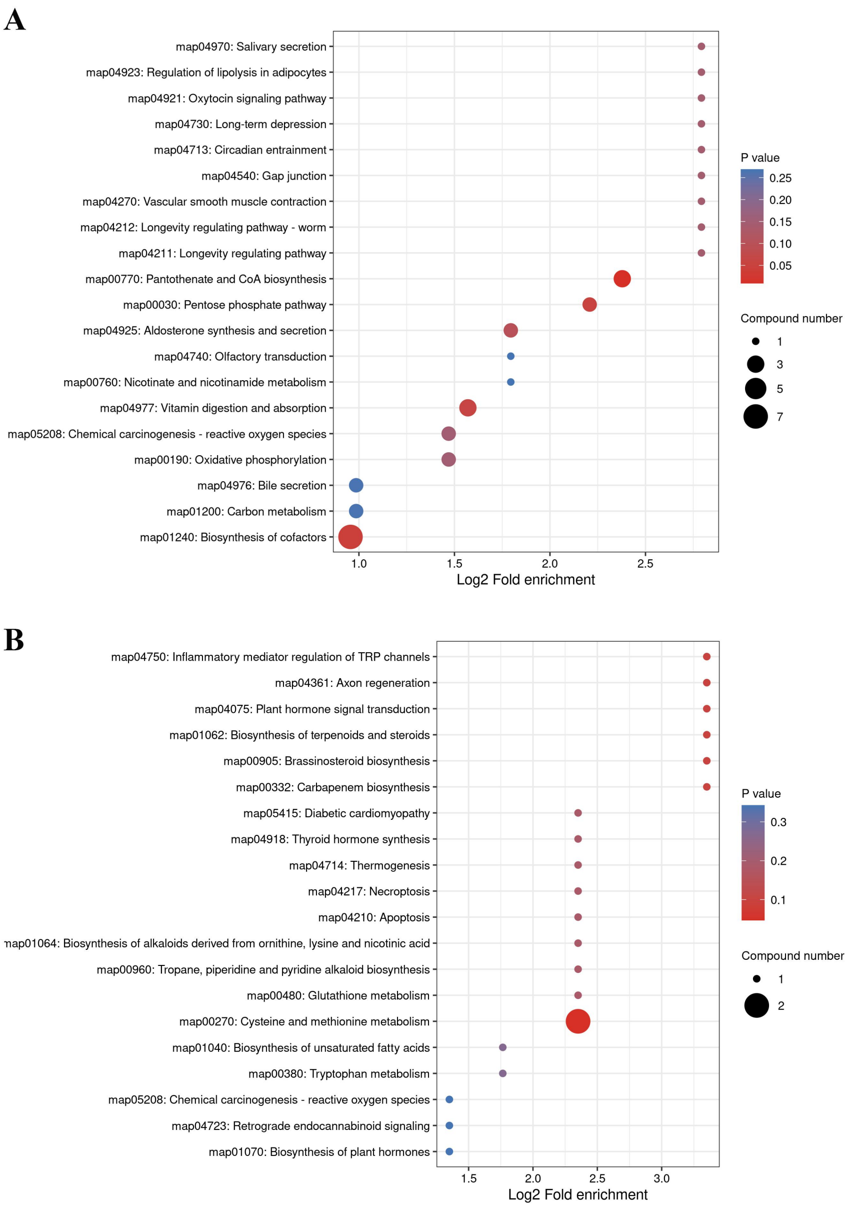

Supplementary Fig. 5. Enrichment pathway analysis between DM and NC groups in negative **(A)** and positive mode **(B)**. Circles represented enriched pathways. Darker circles meant more significant variations in the metabolites in the relevant pathway, whereas the size of the circle indicated the impact. The pathways with P<0.05 were considered as the prominent candidates. The smaller P value indicated the more significantly differential pathway. NC, control db/m group; DM, untreated db/db group.

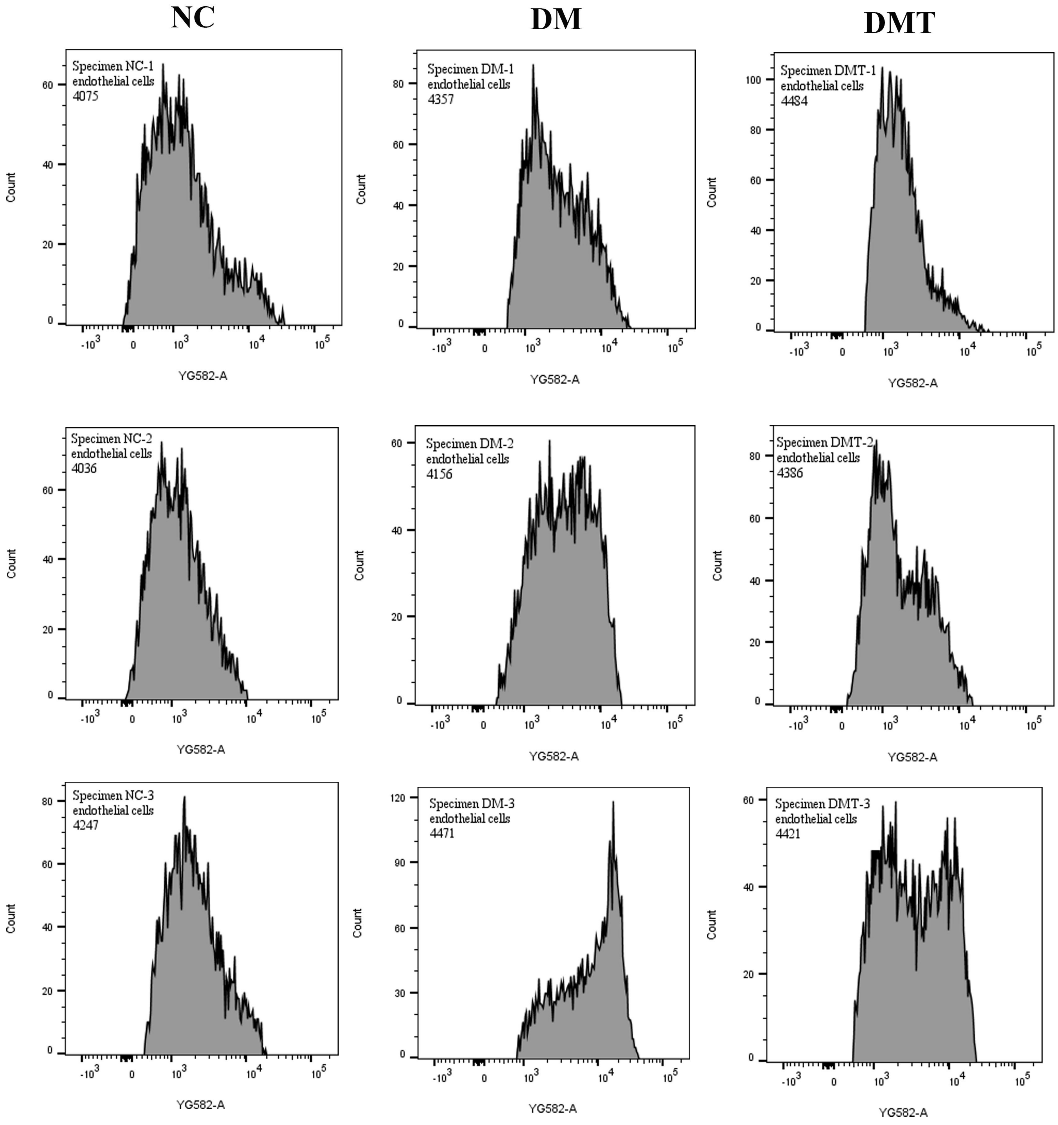

Supplementary Fig. 6. Total of 4000-4500 endothelial cells were purified of each sample by Flow cytometric analysis. NC, control db/m group; DM, untreated db/db group; DMT, human umbilical cord mesenchymal stem cells (hUCMSCs) treated db/db group.

Supplementary Tables

Supplementary Table 1 Primers of Real-Time Quantitative-Polymerase Chain Reaction.

Variables	Forward Sequence (5′-3′)	Reverse Sequence (5′-3′)
CD31	*GACTGAACCTGTCCTGCTCC*	*CACTGTCCGACTTTGAGGCT*
S100a4	*GAGCTGCCCAGCTTCTTG*	*TGCAGGACAGGAAGACACAG*
Tgfb1i1	*TCCCCTGTTCTCCCAAAGC*	*GCCCTGAGGCTGGAAGATG*
Rock1	*AATCGTGTGGGATGCTACCT*	*AAAACCCTCAGTGTGTTGTGC*
NAPE-PLD	*GTCCTCATCAGTCACAACC*	*AGCTCAATCACGTTCTCG*
GAPDH	*GGAGCGAGATCCCTCCAAAAT*	*GGCTGTTGTCATACTTCTCATGG*

CD31, platelet endothelial cell adhesion molecule-1; S100a4, protein S100-A4; Tgfb1i1, transforming growth factor beta-1-induced transcript 1 protein; Rock1, Rho-associated coiled-coil containing protein kinase 1; NAPE-PLD, N-acyl phosphatidylethanolamine-hydrolyzing phospholipase D; GAPDH, glyceraldehyde-3-phosphate dehydrogenase.

Supplementary Table 2 Effects of hUCMSCs on body weight and fasting blood glucose.

Variables	NC (n=6)	DM (n=6)	DMT (n=6)	*F/Welch F*	*P value*
BW (g)					
14 w	32.55 ± 1.57	38.85 ± 4.39**	34.65 ± 4.35	4.562	0.028
18 w	33.60 ± 2.06	31.12 ± 5.60	31.02 ± 6.13	0.527	0.601
22 w	35.38 ± 2.42	26.33 ± 3.15**	28.95 ± 7.08	5.928	0.013
FBG (mmol/L)					
14 w	6.60 ± 2.86	25.77 ± 6.69**	21.23 ± 4.13	25.807	< 0.001
18 w	4.30 ± 0.63	20.87 ± 5.83**	16.53 ± 8.31	12.836	0.001
22 w	5.15 ± 0.63	15.35 ± 3.82**	9.97 ± 3.82#	15.856	< 0.001
GSP (mmol/L)	3.36 ± 0.41	5.21 ± 0.77**	4.55 ± 1.21	7.179	0.006
IL-6 (pg/mL)	30.70 ±15.92	49.45 ± 21.41	45.95 ± 16.52	1.816	0.197
IL-10 (pg/mL)	656.49±244.17	577.05±108.34	1090.34±148.18##	14.725	< 0.001

BW, body weight; FBG, fasting blood glucose; GSP, glycosylated serum protein; IL-6, interleukin-6; IL-10, interleukin-10; ANOVA, one-way analysis of variance; NC, control db/m group; DM, untreated db/db group; DMT, human umbilical cord mesenchymal stem cells (hUCMSCs) treated db/db group.

**P<0.01 compared with NC group; #P<0.05 compared with DM group; ##P<0.01 compared with DM group. All P-values are Bonferroni-adjusted.

Supplementary Table 3 Effects of hUCMSCs on morphology and immunohistological staining.

Variables	NC (n=6)	DM (n=6)	DMT (n=6)	*F/ Welch F*	*P value*
IMT (μm)	54.06 ± 7.56	67.91 ± 5.96**	55.67 ± 7.73#	6.769	0.008
Collagens (mean area)	4.48 ± 1.07	10.89 ± 2.15**	7.49 ± 1.53#	22.821	< 0.001
CD31 (mean IOD)	49.91 ± 10.21	32.71 ± 6.73**	45.51 ± 8.54#	6.456	0.009
Tgfb1i1 (mean IOD)	16.29 ± 4.24	36.99 ± 7.50**	18.95 ± 3.55##	26.308	< 0.001

IMT, intima-media thickness; CD31, platelet/endothelial cell adhesion molecule 1; Tgfb1i1, transforming growth factor beta-1-induced transcript 1 protein; IOD, intensive optical density; NC, control db/m group; DM, untreated db/db group; DMT, human umbilical cord mesenchymal stem cells (hUCMSCs) treated db/db group.
**P<0.01 compared with NC group; #P<0.05 compared with DM group; ##P <0.01 compared with DM group. All P-values are Bonferroni-adjusted.

Supplementary Table 4 Assessment Parameters of OPLS-DA models.

	ESI-		ESI+	
Groups	R2Y	Q2	R2Y (cum)	Q2
DM - NC	0.996	0.664	0.994	0.698
DMT - NC	0.993	0.811	0.998	0.783
DMT - DM	1	0.921	1	0.917

R2Y indicated the rate of model interpretation, and Q2 indicated the model predictive ability.
OPLS-DA, Orthogonal partial least squares discriminate analysis. NC, control db/m group; DM, untreated db/db group; DMT, human umbilical cord mesenchymal stem cells (hUCMSCs) treated db/db group.

Supplementary Table 5 Differential metabolites between DM and NC in negative mode.

No.	ID	Compound name	m/z	rt (s)	VIP	FC	*P value*
1	PTM_808	11,14,17-eicosatrienoic acid, (z,z,z)-	-0.08	10.88	1.52	4.35	0.033
2	PTM_79	stearidonic acid	0.66	9.49	1.51	3.92	0.038
3	PTM_764	19(20)-epdpe	1.44	8.96	1.51	3.41	0.034
4	PTM_624	5(z),11(z),14(z)-eicosatrienoic acid	1.57	11.08	1.43	2.24	0.049
5	PTM_884	1-(1z-octadecenyl)-2-(4z,7z,10z,13z,16z,19z-docosahexaenoyl)-sn-glycero-3-phosphoethanolamine	-1.02	4.97	1.56	0.16	0.025
6	PTM_441	dephospho-coa	0.37	1.62	1.58	16.63	0.012
7	PTM_380	flavine mononucleotide (fmn)	0.15	3.08	1.70	2.41	0.002
8	PTM_199	nicotinamide adenine dinucleotide (nad)	-4.35	0.85	1.47	1.62	0.048
9	PTM_498	cgmp	2.35	1.09	1.54	0.66	0.018
10	PTM_15	uridine monophosphate (ump)	-0.70	1.03	1.70	0.62	0.005
11	PTM_541	adenylosuccinic acid	-0.35	0.76	1.54	0.42	0.018
12	PTM_934	(r)-pantetheine	0.08	2.99	1.58	6.18	0.015
13	PTM_361	glutathione	-0.65	3.11	1.55	4.85	0.033
14	PTM_702	2-methylcitrate	-5.52	0.75	1.70	2.62	0.003
15	PTM_179	o-tyrosine	4.70	2.91	1.42	0.51	0.050
16	PTM_547	acamprosate	0.83	1.74	1.61	0.20	0.021
17	PTM_148	pantothenic acid	3.48	1.77	1.53	3.11	0.022
18	PTM_368	gluconic acid	5.35	0.68	1.50	2.09	0.027
19	PTM_698	2-phenylethyl b-d-glucopyranoside	-0.87	3.42	1.48	0.62	0.037
20	PTM_235	maltopentaose	-0.41	0.86	1.73	0.05	0.001
21	PTM_90	sedoheptulose 7-phosphate	2.33	0.71	1.53	2.14	0.030
22	PTM_305	indolelactic acid	2.86	3.43	1.50	1.89	0.044
23	PTM_429	diprotin a	-8.15	3.19	1.46	0.58	0.044
24	PTM_4	xanthurenic acid	-8.74	2.30	1.59	0.50	0.016
25	PTM_412	emodin 8-glucoside	1.16	3.55	1.66	0.23	0.009
26	PTM_753	2,2'-methylenebis(4-methyl-6-tert-butylphenol)	-0.71	10.37	1.65	0.59	0.004

Fold changes (FC) were acquired as the average levels in DM relative to those in NC. FC>1 indicated up-regulated, FC<1 indicated down-regulated. NC, control db/m group; DM, untreated db/db group. RT, retention time; FC, fold change; VIP, variable influence on projection. All *P*-values are Bonferroni-adjusted.

Supplementary Table 6 Differential metabolites between DM and NC in positive mode.

No.	ID	Name	m/z	rt (s)	VIP	FC	*P value*
1	PTM_1850	diisobutyl phthalate	-0.62	7.59	1.56	0.45	0.034
2	PTM_2053	albuterol	-11.55	4.57	1.68	0.41	0.015
3	PTM_2250	4-methylphenyl octanoate	1.06	7.05	1.54	0.59	0.031
4	PTM_1807	ethyl menthane carboxamide	-0.28	7.82	1.68	0.39	0.007
5	PTM_1045	neryl butyrate	-0.46	5.98	1.75	0.48	0.003
6	PTM_7	zingiberenol	-6.80	9.26	1.72	0.49	0.005
7	PTM_2150	8,11,14-eicosatrienoic acid	-2.67	9.17	1.62	0.45	0.028
8	PTM_2432	2-[octahydro-4,7-dimethyl-1-oxocyclopenta[c]pyran-3-yl]nepetalactam	-7.32	7.04	1.76	0.21	0.002
9	PTM_2711	(e,e)-2,4-decadienoic isobutylamide	1.01	8.18	1.60	0.51	0.026
10	PTM_1948	brassinolide	-5.98	11.35	1.53	0.56	0.048
11	PTM_1762	geranyl acetoacetate	-12.87	7.19	1.61	0.52	0.031
12	PTM_2514	1-palmitoyl-sn-glycero-3-phosphocholine	5.47	10.28	1.55	0.53	0.045
13	PTM_2599	1-heptadecanoyl-sn-glycero-3-phosphocholine	0.68	9.06	1.66	0.54	0.014
14	PTM_2752	(2r)-3-hydroxyisovaleroylcarnitine	1.14	1.64	1.56	2.87	0.026
15	PTM_126	thr-gln	8.91	3.71	1.73	0.32	0.008
16	PTM_1523	l-cysteine-glutathione disulfide	11.32	0.81	1.48	0.51	0.048
17	PTM_1726	glutathione	-4.44	2.5	1.80	7.08	0.001
18	PTM_1870	d-pantetheine 4'-phosphate	1.21	1.74	1.74	16.05	0.005
19	PTM_83	tyrosyl-aspartate	-0.46	1.52	1.51	1.99	0.038
20	PTM_2030	arachidonoyl ethanolamide	-5.37	9.41	1.67	0.65	0.017
21	PTM_2572	1-octadecanamine	0.47	8.98	1.53	0.03	0.041
22	PTM_155	tetradecylamine	-0.01	6.71	1.72	0.41	0.005
23	PTM_189	sphingosine	0.76	7.4	1.63	0.34	0.019
24	PTM_9	xestoaminol c	0.51	6.29	1.77	0.56	0.003
25	PTM_1063	n-oleoylethanolamine	7.75	8.55	1.58	0.34	0.032
26	PTM_2764	(+/-)-[r-(e)]-5-isopropyl-8-methylnona-6,8-dien-2-one	0.21	7.82	1.75	0.40	0.002
27	PTM_2315	3-ketosphingosine	0.17	9.17	1.59	0.61	0.027
28	PTM_2693	(z)-9-cycloheptadecen-1-one	-0.38	8.27	1.47	0.57	0.039
29	PTM_2055	afmk	0.44	2.43	1.61	0.32	0.027
30	PTM_1229	luteinizing hormone releasing hormone human	5.78	2.32	1.47	0.51	0.042
31	PTM_2041	ambronide	0.38	9.68	1.54	0.59	0.028
32	PTM_117	tridemorph	-1.11	11.93	1.69	0.30	0.007
33	PTM_1960	benzyl cinnamate	-11.36	1.35	1.52	0.65	0.036
34	PTM_2618	1-(4-hydroxy-3,5-dimethoxyphenyl)-7-(4-hydroxy-3-methoxyphenyl)-3,5-heptanediol	-17.37	4.88	1.58	0.43	0.032

Fold changes (FC) were calculated as the average levels in DM relative to those in

NC. FC>1 indicated up-regulated, FC<1 indicated down-regulated. NC, control db/m group; DM, untreated db/db group. RT, retention time; FC, fold change; VIP, variable influence on projection. RT, retention time; FC, fold change; VIP, variable influence on projection. All *P*-values are Bonferroni-adjusted.

Supplementary Table 7 Effects of hUCMSCs on metabolites altered in DM.

No.	ID	Name	m/z	rt (s)	mode	VIP	FC	*P value*
1	PTM_90	sedoheptulose 7-phosphate	2.33	0.71	ESI-	1.46	0.37	0.001
2	PTM_148	pantothenic acid	3.48	1.77	ESI-	1.44	0.58	0.002
3	PTM_934	(r)-pantetheine	0.08	2.99	ESI-	1.39	0.11	0.008
4	PTM_702	2-methylcitrate	-5.52	0.75	ESI-	1.42	0.41	0.004
5	PTM_441	dephospho-coa	0.37	1.62	ESI-	1.32	0.09	0.021
6	PTM_380	flavine mononucleotide (fmn)	0.15	3.08	ESI-	1.41	0.29	0.004
7	PTM_764	19(20)-epdpe	1.44	8.96	ESI-	1.26	0.30	0.034
8	PTM_79	stearidonic acid	0.66	9.49	ESI-	1.41	0.10	0.005
9	PTM_808	11,14,17-eicosatrienoic acid, (z,z,z)-	-0.08	10.88	ESI-	1.39	0.09	0.006
10	PTM_624	5(z),11(z),14(z)-eicosatrienoic acid	1.57	11.08	ESI-	1.38	0.24	0.008
11	PTM_4	xanthurenic acid	-8.74	2.30	ESI-	1.23	2.04	0.042
12	PTM_235	maltopentaose	-0.41	0.86	ESI-	1.40	6.16	0.005
13	PTM_698	2-phenylethyl b-d-glucopyranoside	-0.87	3.42	ESI-	1.45	3.18	0.001
14	PTM_541	adenylosuccinic acid	-0.35	0.76	ESI-	1.42	2.84	0.004
15	PTM_15	uridine monophosphate (ump)	-0.70	1.03	ESI-	1.40	1.68	0.006
16	PTM_884	1-(1z-octadecenyl)-2-(4z,7z,10z,13z,16z,19z-docosahexaenoyl)-sn-glycero-3-phosphoethanolamine	-1.02	4.97	ESI-	1.24	4.16	0.042
17	PTM_1870	d-pantetheine 4'-phosphate	1.21	1.74	ESI+	1.28	0.14	0.022
18	PTM_1850	diisobutyl phthalate	-0.62	7.59	ESI+	1.31	2.38	0.014
19	PTM_2053	albuterol	-11.55	4.57	ESI+	1.43	5.15	0.000
20	PTM_1807	ethyl menthane carboxamide	-0.28	7.82	ESI+	1.42	2.08	0.001
21	PTM_1045	neryl butyrate	-0.46	5.98	ESI+	1.45	2.56	0.000
22	PTM_7	zingiberenol	-6.80	9.26	ESI+	1.44	2.02	0.000
23	PTM_2150	8,11,14-eicosatrienoic acid	-2.67	9.17	ESI+	1.22	2.91	0.039
24	PTM_2432	2-[octahydro-4,7-dimethyl-1-oxocyclopenta[c]pyran-3-yl] nepetalactam	-7.32	7.04	ESI+	1.34	3.34	0.008
25	PTM_2711	(e,e)-2,4-decadienoic isobutylamide	1.01	8.18	ESI+	1.34	2.66	0.009

26	PTM_1948	brassinolide	-5.98	11.35	ESI+	1.43	3.83	0.001
27	PTM_126	thr-gln	8.91	3.71	ESI+	1.41	3.37	0.002
28	PTM_1523	l-cysteine-glutathione disulfide	11.32	0.81	ESI+	1.42	5.43	0.001
29	PTM_2030	arachidonoyl ethanolamide	-5.37	9.41	ESI+	1.42	2.45	0.001
30	PTM_2572	1-octadecanamine	0.47	8.98	ESI+	1.40	6.17	0.002
31	PTM_155	tetradecylamine	-0.01	6.71	ESI+	1.44	5.25	0.000
32	PTM_189	sphingosine	0.76	7.4	ESI+	1.38	4.44	0.003
33	PTM_9	xestoaminol c	0.51	6.29	ESI+	1.44	3.38	0.000
34	PTM_2764	(+/-)-[r-(e)]-5-isopropyl-8-methylnona-6,8-dien-2-one	0.21	7.82	ESI+	1.37	2.08	0.006
35	PTM_2315	3-ketosphingosine	0.17	9.17	ESI+	1.37	2.05	0.005
36	PTM_2693	(z)-9-cycloheptadecen-1-one	-0.38	8.27	ESI+	1.41	2.53	0.002
37	PTM_2041	ambronide	0.38	9.68	ESI+	1.35	2.02	0.008
38	PTM_117	tridemorph	-1.11	11.93	ESI+	1.36	2.92	0.006

Fold changes (FC) were calculated as the average levels in DMT relative to those in DM. FC>1 indicated up-regulated, FC<1 indicated down-regulated. hUCMSCs, human umbilical cord mesenchymal stem cells; DM, untreated db/db group; DMT, human umbilical cord mesenchymal stem cells (hUCMSCs) treated db/db group. RT, retention time; FC, fold change; VIP, variable influence on projection. All *P*-values are Bonferroni-adjusted.

Supplementary Table 8 Effects of hUCMSCs on endothelial proteins altered in DM presented in volcano figures and marked out in PPI.

No.	ID	Protein Name	Gene name	DM/ NC	*P value*	DMT/ DM	*P value*
1	P12382	ATP-dependent 6-phosphofructokinase	Pfkl	10.34	<0.001	0.17	0.007
2	P06745	Glucose-6-phosphate isomerase	Gpi	1.99	<0.001	0.56	<0.001
3	P09411	Phosphoglycerate kinase 1	Pgk1	1.87	<0.001	0.44	0.001
4	Q9DBJ1	Phosphoglycerate mutase 1	Pgam1	3.98	0.001	0.25	0.001
5	P52480	Pyruvate kinase PKM	Pkm	1.84	<0.001	0.55	<0.001
6	Q9JLJ2	4-trimethylaminobutyraldehyde dehydrogenase	Aldh9a1	2.37	0.043	0.49	0.001
7	P28474	Alcohol dehydrogenase class-3	Adh5	4.48	<0.001	0.52	0.018
8	P07091	Protein S100-A4	S100a4	5.58	<0.001	0.41	<0.001
9	Q62219	Transforming growth factor beta-1-induced transcript 1 protein	Tgfb1i1	4.31	<0.001	0.55	0.001
10	Q99JY0	Trifunctional enzyme subunit beta	Hadhb	0.61	<0.001	1.54	<0.001
11	Q06185	ATP synthase subunit e	Atp5me	0.43	<0.001	1.65	<0.001
12	Q9DCX2	ATP synthase subunit d	Atp5pd	0.50	<0.001	1.54	<0.001
13	Q6P8J7	Creatine kinase S-type	Ckmt2	0.12	0.001	6.22	0.002
14	Q8K2B3	Succinate dehydrogenase	Sdha	0.55	<0.001	1.69	<0.001
15	Q9Z2Z6	Mitochondrial carnitine/acylcarnitine carrier protein	Slc25a20	0.55	0.019	1.98	0.001
16	P00397	Cytochrome c oxidase subunit 1	Mtco1	0.36	<0.001	1.85	0.001
17	P00405	Cytochrome c oxidase subunit 2	Mtco2	0.40	<0.001	1.51	0.006
18	Q9CZ13	Cytochrome b-c1 complex subunit 1	Uqcrc1	0.47	<0.001	1.91	<0.001
19	Q9DB77	Cytochrome b-c1 complex subunit 2	Uqcrc2	0.45	<0.001	1.84	<0.001
20	Q9ESL4	Mitogen-activated protein kinase kinase kinase 20	Map3k20	2.97	<0.001	0.32	0.001
21	O35598	Disintegrin and metalloproteinase domain-containing protein 10	Adam10	2.16	0.008	0.42	0.008
22	A2ARA8	Integrin alpha-8	Itga8	2.29	<0.001	0.51	<0.001
23	P70335	Rho-associated protein kinase 1	Rock1	5.56	<0.001	0.49	0.015
24	Q99MS7	EH domain-binding protein 1-like protein 1	Ehbp1l1	0.39	<0.001	1.86	0.001
25	Q9JK53	Prolargin	Prelp	0.06	<0.001	10.84	<0.001
26	Q06890	Clusterin	Clu	0.35	0.041	2.44	0.040
27	P47857	ATP-dependent 6-phosphofructokinase, muscle type	Pfkm	2.23	<0.001	0.38	0.001
28	Q9WUA3	ATP-dependent 6-phosphofructokinase, platelet type	Pfkp	3.00	<0.001	0.22	<0.001
29	P12787	Cytochrome c oxidase subunit 5A	Cox5a	0.38	<0.001	2.16	0.001
30	P19536	Cytochrome c oxidase subunit 5B	Cox5b	0.52	<0.001	1.97	<0.001

31	Q9CPQ1	Cytochrome c oxidase subunit 6C	Cox6c	0.39	<0.001	2.18	0.005
32	P56392	Cytochrome c oxidase subunit 7A1	Cox7a1	0.05	<0.001	17.69	<0.001
33	P17665	Cytochrome c oxidase subunit 7C	Cox7c	0.32	<0.001	2.43	<0.001
34	Q9CQ69	Cytochrome b-c1 complex subunit 8	Uqcrq	0.38	<0.001	2.31	<0.001
35	Q9CR68	Cytochrome b-c1 complex subunit Rieske	Uqcrfs1	0.44	0.001	2.02	<0.001
36	Q9D855	Cytochrome b-c1 complex subunit 7	Uqcrb	0.46	<0.001	1.80	<0.001
37	P99028	Cytochrome b-c1 complex subunit 6	Uqcrh	0.45	<0.001	1.54	0.044
38	Q08093	Calponin-2	Cnn2	12.38	0.002	0.58	0.001
39	P62869	Elongin-B	Elob	2.23	<0.001	0.56	0.004
40	P83940	Elongin-C	Eloc	5.33	0.001	0.34	<0.001
41	P62754	40S ribosomal protein S6	Rps6	1.70	0.003	0.58	0.011

hUCMSCs, human umbilical cord mesenchymal stem cells; PPI, protein-protein interactions. NC, control db/m group; DM, untreated db/db group; DMT, human umbilical cord mesenchymal stem cells (hUCMSCs) treated db/db group. All *P*-values are Bonferroni-adjusted.

Supplementary methods and materials

1.1. Materials

Anti-mouse platelet/endothelial cell adhesion molecule 1 (CD31) antibody (28083-1-AP), anti-mouse transforming growth factor beta-1-induced transcript 1 protein (Tgfb1i1) antibody (10565-1-AP), anti-mouse disintegrin and metalloproteinase domain-containing protein 10 (Adam10) antibody (25900-1-AP), anti-mouse Rho-associated protein kinase 1 (Rock1) antibody (16105-1-AP), anti-mouse Protein S100-A4 (S100a4) antibody (16105-1-AP) and anti-mouse transgelin (Tagln) antibody (10493-1-AP) were purchased from Proteintech Group (Rosemont, USA). Anti-mouse mitogen-activated protein kinase kinase kinase 20 (Map3k20) antibody (TA366749S) was purchased from OriGene Technologies (Maryland, USA). Anti-mouse Integrin α 8 (Itga8) antibody (sc-365798) was purchased from Santa Cruz Biotechnology (Santa Cruz, USA). PE anti-mouse CD31 antibody (160204) was procured from BioLegend (California, USA).

1.2. Preparation of hUCMSCs

Fresh umbilical cords were obtained from informed, consenting mothers at the Qilu Hospital of Shandong University (China) and handled immediately. The cords were rinsed twice with phosphate-buffered saline (PBS) mixed with penicillin and streptomycin (pen/strep; Gibco, Carlsbad, CA), and the vessels were eliminated. The washed cords were then cut as small as possible and attached to the substrate of culture plates separately. Subsequently, they were incubated in stem cell culture medium (Gibco, Carlsbad, CA) at 37 °C with 5% CO2. The medium was refreshed regularly until fibroblast-like cell colonies observed after about 7~10 days. The cells were then trypsinized (Gibco) and passaged. The F3-7 generation cells were applied for the experiments. And the hUCMSCs were identified according to the criteria suggested by the International Society for Cellular Therapy [11], with surface marker analysis by flow cytometry, as well as bone formation, fat induction, and chondrogenic differentiation using Alizarin Red, Oil Red O, and Alcian Blue staining, respectively. The experimental protocol was approved by the Shandong University Ethics Committee.

1.3. Flow cytometry and endothelial cell sorting

Perivascular adipose and connective tissues were isolated and removed from the vascular tissue before dissociation and single-cell analysis. The aortic tissues were cut into 0.5 mm2 pieces and treated with enzyme solution (1mg/ml collagenase I, 0.5 mg/ml Elastase, 1mg/ml DNase I) in 37℃ water bath shaking at 100 rpm for 40 min. Digestion was terminated by 1× PBS containing 10% fetal bovine serum (FBS) and pipetted for 5-10 times. The cell suspension was filtered through 30um stacked cell strainer and then centrifuged at 300g and 4°C for 5 min. The cell pellet was resuspended with 100ul 1× PBS (0.04% BSA). The overall cell viability was more than 90% examined by trypan blue exclusion.

The single cell suspension was stained with PE CD31 on ice for 20 min, washed twice and resuspended with 5ml of fluorescence-activated cell sorting (FACS) buffer.

Stained cells were sorted by FACSAriaTM III Cell sorter (BD Biosciences) by a 70 μm nozzle.
To obtain aortic endothelial cells of mice, FACS events were screened through the following nested gates: 1) plotting forward scatter area (FSC-A) against side scatter area (SSC-A) to exclude large clusters and small debris; 2) singlets-set by excluding the margins of SSC-H and SSC-A width plot; 3) EC, by gating CD31-positive population, and 4) aortic ECs, inferred from the CD31 histogram.

1.4. Untargeted metabolomics

1.4.1. Sample preparation and metabolite extraction

5mg of aortic samples were homogenized with 50 μL of H2O. 200 μL methanol/acetonitrile (MeOH/ACN, 1:1, v/v) solvent were mixed, vortexed and sonicated with the solution for metabolite extraction. To each sample, 50 μL of ACN/H2O (1:1, v/v) mixture was added for dryness, and pulverized using ultrasonic wave for 10 min on ice. After centrifugation at 12,000 g at 4°C for 15 min, the supernatant was collected and kept in -80℃ for LC/MS analysis.

1.4.2. HPLC-Q-TOF MS analysis

HPLC-Q-TOF MS analysis was applied using a 1290 Infinity Ultra-high Performance Liquid Chromatography system (Agilent Technologies, Palo Alto, CA, USA) attached to a Triple TOF 6600 system (AB/SCIEX, Framingham, MA, USA).

Chromatographic separation was performed on a Waters ACQUITY UPLC BEH Amide 1.7 μm 2.1 mm × 100 mm column, with the injection volume of 2 μL and flow rate of 0.3 mL/min at 25℃. The mobile phase consisted of A (water and 0.1% formic acid) and B (acetonitrile and 0.1% formic acid).

For MS experiments, this study was proceeded in electrospray ionization (ESI) positive and negative ion modes. The ESI source situations after chromatographic separation were as follows: Ion Source Gas1 (Gas1): 60psi, Ion Source Gas2 (Gas2): 60psi, Curtain gas (CUR): 30psi, source temperature: 600 ° C, Ion Sapary Voltage Floating (ISVF) ± 5500V (± ESI); TOF MS scan m/z range: 60-1000Da, product ion scan m/z range: 25-1000Da, TOF MS scan accumulation time 0.20s/spectra, product ion scan accumulation time 0.05s/spectra; the secondary mass spectra were obtained by information dependent acquisition (IDA) in the peak intensity screening mode, Declustering potential (DP): ± 60V (± ESI), collision Energy: 35 ± 15 eV, IDA was set to dynamically exclude isotope ions within 4Da and gather 10 fragment maps per scan.

1.4.3. Data processing

Firstly, the quality of the mass spectrometry data were evaluated by quality control samples (QC) and principal component analysis (PCA).
After sum-normalization, Relative Standard Deviation (RSD) was used to assess the robustness of the model. The variable importance in the projection (VIP) values obtained from a supervised model of orthogonal partial least squares discrimination

analysis (OPLS-DA) were applied to describe the infuence and interpretation ability of each metabolite, thereby to find metabolic markers. Student's t- test and fold change analysis (FC) were proceeded between two groups. VIP > 1 and P value < 0.05 were used to discover significantly different metabolites. For more than 3 groups, Analysis of Variance (Anova) analysis was performed by default. Finally, MetaboAnalyst 4.0 (http://www.metaboanalyst.ca) and KEGG (http://www.genome.jp/kegg) were adopted to cluster analysis and metabolic pathway analysis on differential metabolites.

1.5. Proteomics Study

The timsTOF Pro mass spectrometer was applied for as a qualitative and quantitative analysis of differential proteins called the 4D (ion mobility, m/z, retention time, and intensity) label free proteomics for single cell type on basis of trapped ion mobility (TIMS) separation and parallel cumulative serial fragmentation (PASEF) scanning. Protein preparation, LC-MS/MS analysis, and bioinformatics analysis contributed to the test.

1.5.1. Protein preparation

Each sample of aortic endothelial cells sorted by FACS of mice in NC, DM, and DMT group was mixed with 10 μL digestion buffer (1% SDC) for lysis and protein extraction. The mixture underwent non-contact ultrasonication for 3 minutes and was then heated at 95°C for 10 minutes. After cooling to room temperature, trypsin was added to achieve a final concentration of 10 ng/μL, and the samples were digested overnight at 37°C. Dithiothreitol (DTT) was added to a final concentration of 5 mM, and the samples were reduced at 56°C for 30 minutes. Following this, iodoacetamide (IAA) was introduced to a final concentration of 11 mM, and the samples were incubated in the dark at room temperature for 15 minutes. Ultimately, peptides were desalted using Ziptip (Merck, ZTC18S096), according to the manufacturer's instructions.

1.5.2. LC-MS/MS analysis

Liquid crystal tandem mass spectrometry (LC-MS/MS) analysis was proceeded on a timsTOF Pro mass spectrometer (Bruker Daltonics, Bremen, Germany) that was coupled to Nanoelute (Bruker Daltonics, Bremen, Germany). The tryptic peptides were processed onto a reversed-phase analytical column (20-cm length, 50 μm i.d.) using buffer A (0.1% formic acid, 2% acetonitrile/ in water) and divided using a linear gradient of buffer B (0.1% formic acid in acetonitrile) at a flow rate of 450 nL/min. The mass spectrometer (MS) was performed with MS/MS scan range from 100 to 1700 m/z in parallel accumulation serial fragmentation (PASEF) mode. Precursors with charge states 0 to 5 were used for fragmentation, and 3 PASEF-MS/MS scans were adopted per cycle. The dynamic exclusion was set to 30 s.

1.5.3. Bioinformatic analysis of proteins

The MS raw data of each sample were processed by the MaxQuant (v.1.6.15.0) software to identify and quantify the peptides. Tandem mass spectra were searched against the human SwissProt database (20389 entries) concatenated with reverse decoy database. Trypsin/P was specified as cleavage enzyme allowing up to 2 missing cleavages. The mass tolerance for precursor ions was set as 20 ppm in First and Main search, as well as for fragment ions. Carbamidomethyl on Cys was selected as fixed modification, and acetylation on protein N-terminal and oxidation on Met were selected as variable modifications. The false discovery rate (FDR) thresholds was applied as ≤1%. Only ratios with P-values <0.05 and fold changes >1.5 were regarded as significant.

The protein sequences of the selected differentially abundant proteins were searched locally in the NCBI BLAST+ client software (ncbi-blast-2.2.28 + − win32.exe) and InterProScan was applied to discover homolog sequences. Then, gene ontology (GO) terms were mapped and sequences were annotated by the software program Blast2GO. The GO annotation results were plotted by R scripts. And then, the proteins were BLAST searched in the online Kyoto Encyclopedia of Genes and Genomes (KEGG) database (http://geneonto logy.org/) to retrieve their KEGG orthology identifications and subsequently mapped to pathways in the KEGG database. Enrichment analysis was performed using Fisher's exact test, recognizing all quantified proteins as the background dataset. Benjamini Hochberg correction for multiple comparisons was also produced to adjust the calculated P-values. Only functional categories and pathways with P values <0.05 were recognized as significant. The protein–protein interaction (PPI) information of the studied proteins was obtained from the IntAct molecular interaction database (http://www.ebi.ac.uk/intact/) through gene symbols or STRING software version 11.0 (http://string-db.org/). The results were downloaded in the XGMML format and imported into Cytoscape software (http://www. cytoscape.org/, version 3.4.0) for visualization and further analysis of functional protein-protein interaction networks.

1.6. Proteomics Study

The timsTOF Pro mass spectrometer was applied for as a qualitative and quantitative analysis of differential proteins called the 4D (ion mobility, m/z, retention time, and intensity) label free proteomics for single cell type on basis of trapped ion mobility (TIMS) separation and parallel cumulative serial fragmentation (PASEF) scanning. Protein preparation, LC-MS/MS analysis, and bioinformatics analysis contributed to the test.

1.6.1. Protein preparation

Each sample of aortic endothelial cells sorted by FACS of mice in NC, DM, and DMT group was mixed with 10 μL digestion buffer (1% SDC) for lysis and protein extraction. The mixture underwent non-contact ultrasonication for 3 minutes and was then heated at 95°C for 10 minutes. After cooling to room temperature, trypsin was added to achieve a final concentration of 10 ng/μL, and the samples were digested overnight at 37°C. Dithiothreitol (DTT) was added to a final concentration of 5 mM,

and the samples were reduced at 56°C for 30 minutes. Following this, iodoacetamide (IAA) was introduced to a final concentration of 11 mM, and the samples were incubated in the dark at room temperature for 15 minutes. Ultimately, peptides were desalted using Ziptip (Merck, ZTC18S096), according to the manufacturer's instructions.

1.6.2. LC-MS/MS analysis

Liquid crystal tandem mass spectrometry (LC-MS/MS) analysis was proceeded on a timsTOF Pro mass spectrometer (Bruker Daltonics, Bremen, Germany) that was coupled to Nanoelute (Bruker Daltonics, Bremen, Germany). The tryptic peptides were processed onto a reversed-phase analytical column (20-cm length, 50 µm i.d.) using buffer A (0.1% formic acid, 2% acetonitrile/ in water) and divided using a linear gradient of buffer B (0.1% formic acid in acetonitrile) at a flow rate of 450 nL/min. The mass spectrometer (MS) was performed with MS/MS scan range from 100 to 1700 m/z in parallel accumulation serial fragmentation (PASEF) mode. Precursors with charge states 0 to 5 were used for fragmentation, and 3 PASEF-MS/MS scans were adopted per cycle. The dynamic exclusion was set to 30 s.

1.6.3. Bioinformatic analysis of proteins

The MS raw data of each sample were processed by the MaxQuant (v.1.6.15.0) software to identify and quantify the peptides. Tandem mass spectra were searched against the human SwissProt database (20389 entries) concatenated with reverse decoy database. Trypsin/P was specified as cleavage enzyme allowing up to 2 missing cleavages. The mass tolerance for precursor ions was set as 20 ppm in First and Main search, as well as for fragment ions. Carbamidomethyl on Cys was selected as fixed modification, and acetylation on protein N-terminal and oxidation on Met were selected as variable modifications. The false discovery rate (FDR) thresholds was applied as ≤1%. Only ratios with *P*-values <0.05 and fold changes >1.5 were regarded as significant.

The protein sequences of the selected differentially abundant proteins were searched locally in the NCBI BLAST+ client software (ncbi-blast-2.2.28 + − win32.exe) and InterProScan was applied to discover homolog sequences. Then, gene ontology (GO) terms were mapped and sequences were annotated by the software program Blast2GO. The GO annotation results were plotted by R scripts. And then, the proteins were BLAST searched in the online Kyoto Encyclopedia of Genes and Genomes (KEGG) database (http://geneonto logy.org/) to retrieve their KEGG orthology identifications and subsequently mapped to pathways in the KEGG database. Enrichment analysis was performed using Fisher’s exact test, recognizing all quantified proteins as the background dataset. Benjamini Hochberg correction for multiple comparisons was also produced to adjust the calculated *P*-values. Only functional categories and pathways with *P* values <0.05 were recognized as significant. The protein–protein interaction (PPI) information of the studied proteins was obtained from the IntAct molecular interaction database (http://www.ebi.ac.uk/intact/) through gene symbols or STRING software version 11.0 (http://string-db.org/). The results were downloaded in

the XGMML format and imported into Cytoscape software (http://www. cytoscape.org/, version 3.4.0) for visualization and further analysis of functional protein-protein interaction networks.
